# Throughout *in vitro* first spermatogenic wave: Next-generation sequencing gene expression patterns of fresh and cryopreserved prepubertal mice testicular tissue explants

**DOI:** 10.3389/fendo.2023.1112834

**Published:** 2023-03-17

**Authors:** Ludovic Dumont, Hélène Lopez Maestre, Frédéric Chalmel, Louise Huber, Aurélie Rives-Feraille, Laura Moutard, Frédérique Bateux, Christine Rondanino, Nathalie Rives

**Affiliations:** ^1^ Univ Rouen Normandie, INSERM, NORDIC UMR 1239 – Team Adrenal and Gonadal Pathophysiology (AGoPath), Rouen, France; ^2^ Normandie, Institute for Research and Innovation in Biomedicine (IRIB), Rouen, France; ^3^ Univ Rouen Normandie, INSERM, PANTHER UMR 1234, Rouen, France; ^4^ Institut Pasteur, Hub de Bioinformatique et Biostatistique – Département Biologie Computationnelle, USR 3756, CNRS, Paris, France; ^5^ University of Rennes 1, Inserm U1085-IRSET, Rennes, France; ^6^ Rouen University Hospital, Biology of Reproduction-CECOS laboratory, Rouen, France

**Keywords:** cryopreservation, *in vitro* spermatogenesis, mice, RNA-Seq, testis, transcriptomic

## Abstract

**Introduction:**

Suitable cryopreservation procedures of pre-pubertal testicular tissue associated with efficient culture conditions are crucial in the fields of fertility preservation and restoration. *In vitro* spermatogenesis remains a challenging technical procedure to undergo a complete spermatogenesis.The number of haploid cells and more specifically the spermatic yield produced *in vitro* in mice is still extremely low compared to age-matched *in vivo* controls and this procedure has never yet been successfully transferred to humans.

**Methods:**

To evaluate the impact of in vitro culture and freezing procedure, pre-pubertal testicular mice testes were directly cultured until day 4 (D4), D16 and D30 or cryopreserved by controlled slow freezing then cultured until D30. Testes composed of a panel of 6.5 dpp (days postpartum), 10.5 dpp, 22.5 dpp, and 36.5 dpp mice were used as *in vivo* controls. Testicular tissues were assessed by histological (HES) and immunofluorescence (stimulated by retinoic acid gene 8, STRA8) analyses. Moreover, a detailed transcriptome evaluation study has been carried out to study the gene expression patterns throughout the first *in vitro* spermatogenic wave.

**Results:**

Transcriptomic analyses reveal that cultured tissues expression profiles are almost comparable between D16 and D30; highlighting an abnormal kinetic throughout the second half of the first spermatogenesis during *in vitro* cultures. In addition, testicular explants have shown dysregulation of their transcriptomic profile compared to controls with genes related to inflammation response, insulin-like growth factor and genes involved in steroidogenesis.

**Discussion:**

The present work first shows that cryopreservation had very little impact on gene expression in testicular tissue, either directly after thawing or after 30 days in culture. Transcriptomic analysis of testis tissue samples is highly informative due to the large number of expressed genes and identified isoforms. This study provides a very valuable basis for future studies concerning *in vitro* spermatogenesis in mice.

## Introduction

Spermatogenesis is a tightly regulated and dynamic process of spermatogonial stem cells differentiation which takes place into the seminiferous tubules in the testis. This process requires the orchestrated regulation of mitotic development of spermatogonia and meiotic process of spermatocytes followed by their differentiation in spermatozoa (1). Spermatogenesis is a complex cellular process to produce spermatozoa with germ cell proliferation and differentiation (2). The first wave of spermatogenesis in mice is initiated only a few days after birth and proceeds in a synchronized manner. This entire process, taking ~36 days, consists of three phases: proliferation of spermatogonia, meiotic division of spermatocytes, and morphological change of spermatids. The progression of spermatogenesis is not germ cell autonomous but definitely requires communication between germ and testicular somatic cells, particularly Leydig (3, 4) and Sertoli (5, 6) cells. Steroidogenesis, the biological process by which steroids are generated from cholesterol and changed into other steroids, is a key hormonal process that needs to be intact for proper testicular function (7). In order to have a complete and efficient spermatogenesis, the steroidogenesis has to be regulated at multiple levels, principally by transcription of genes encoding steroidogenic enzymes and co-factors, and by their post-translational modification. In testicular tissue, gene expressions during spermatogenesis is highly orchestrated and strictly regulated at transcriptional and post-transcriptional level. Major periods of expressional change occur during the first few days after birth (0 to 6 d*pp*), at the beginning of meiosis (12 to 14 d*pp*), and along with the appearance of haploid gametes (around 20 d*pp*) (8).

Testicular tissue cryopreservation has been introduced as an advantageous technique for fertility preservation in boys suffering from cancer (9, 10). Because of impairment of human testicular grafting (*e*.*g*., inadequate oxygen, nutrient supplies) (11, 12), dysregulation of angiogenesis-specific signaling (13), and risk of contamination by malignant cells (14), *in vitro* tissue culture has been proposed as an alternative procedure to restore fertility (11). Some teams, including ours, have succeeded in reproducing all stages of the spermatogenesis from spermatogonia to functional sperms *in vitro* by an organ culture of fresh (15, 16) and frozen-thawed neonatal mouse testes (17, 18). In addition, functional flagellated elongated spermatids have been obtained after *in vitro* spermatogenesis from fresh (15) and cryopreserved (19) pre-pubertal testicular tissues. However, the number of haploid cells and more specifically the spermatic yield produced *in vitro* in mice is still extremely low compared to age-matched *in vivo* controls and this procedure has never yet been successfully transferred to humans.

Spermatogenesis was studied from a transcriptome perspective in the physiological first wave of spermatogenesis on mice model (20). To date, very few studies have investigated the first wave of spermatogenesis *in vitro*. Two recent studies conducted by microarray analysis revealed that the deficiency of germ cell differentiation and the immense immune reaction are major abnormalities in the cultured testis tissues (21, 22). Furthermore, the impact of freezing on testicular tissue as well as of organotypic culture of fresh or frozen tissue has been never studied from the point of view of gene expression in testicular tissue. In this study, we profile cells from the first spermatogenic wave to confidently assess the maturation of testicular tissue throughout the developmental trajectory, where cells have only progressed to a defined developmental stage. This allows us to identify the transcriptomic expression of cells at the most advanced stage of *in vitro* cultured seminiferous tubules in comparison with age-matched *in vivo* controls.

**Figure 1:**
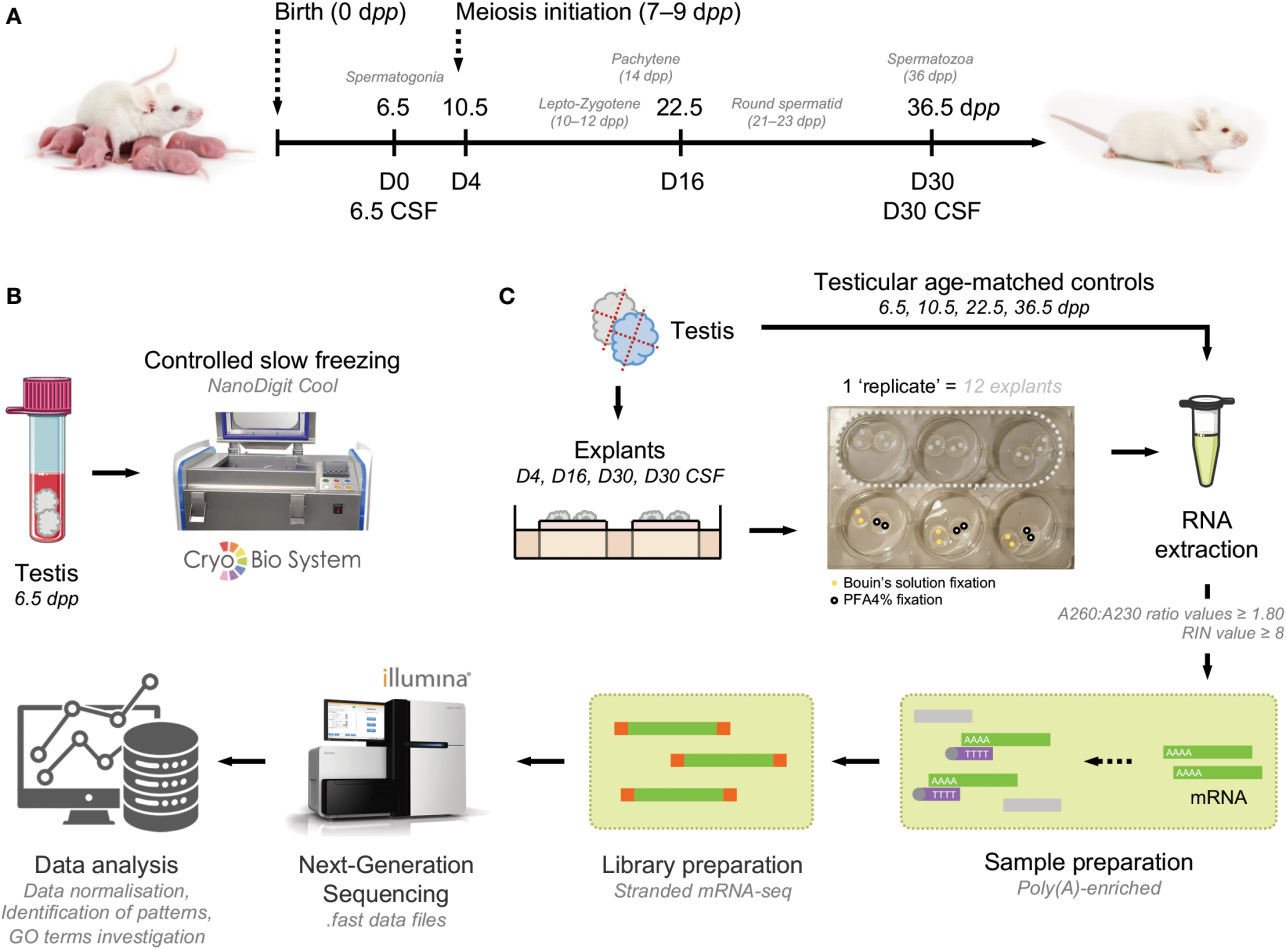
Flow chart of the study design. **(A)** Testicular fragments from 6.5 d*pp* mice were cultured from fresh or frozen-thawed testicular tissue with a medium composed of α-MEM, KSR, gentamycin, and Rol. These cultures were stopped at key time points of the first spermatogenic wave: D4, D16, and D30 for fresh tissue in order to evaluate the impact of *in vitro* culture and stopped at D30 for frozen-thawed tissue in order to evaluate the impact of cryopreservation procedure. Testes from mice of 6.5, 10.5, 22.5, and 36.5 d*pp* correspond to respective age-matched *in vivo* controls. **(B)** Frozen 6.5 d*pp* testicular tissue were cryopreserved by a controlled slow freezing procedure. **(C)** Samples were cut into 4 fragments of 0.75 mm^3^ and cultured onto a pair of agarose gels. Samples were fixed in Bouin’s solution for histological analyses (n=3×6 fragments per condition) and PFA4% for STRA8 immunofluorescence (n=3×6 fragments per condition). Then, in order to perform transcript analysis, RNAs from testicular explants (n=3×6 fragments per condition) as well as their respective *in vivo* controls (n=3×6 fragments per condition) were extracted. Sample and library preparations were carried out before illumina sequencing. Finally, a global genes bioinformatics analysis followed by a differential expression study and an annotation by enrichment with defined gene sets were performed. α-MEM, minimum essential medium Eagle; d*pp*, days *postpartum*; GO, gene ontology; KSR, knockout serum replacement; PFA, paraformaldehyde; Rol, retinol (vitamin A); RIN, RNA integrity number.

In this study, histological analyses were coupled with the expression of RNA-Seq analysis to analyze in depth the first wave of *in vitro* spermatogenesis. Since proper development of male germ cells requires correct function of testicular somatic cells and differences in gene expression may be affected by cell-cell interactions, the study of testicular tissue is a desirable sample type for investigation of gene expression during spermatogenesis. We attempted to characterize the first wave of spermatogenesis *in vivo* and *in vitro* at key time points by comparing the transcriptomes of *in vivo* testicular tissues and *in vitro* fresh and frozen testicular explants. To evaluate the impact of *in vitro* culture and freezing procedure, pre-pubertal testicular mice testes were (i) directly cultured until D4, D16 and D30 or (ii) cryopreserved by controlled slow freezing (CSF) then cultured until D30. Testes composed of a panel of 6.5 d*pp* (before meiosis initiation), 10.5 d*pp* (lepto-zygotene spermatocyte I stage), 22.5 d*pp* (round spermatid stage) and 36.5 d*pp* (elongated spermatid stage) mice were used as *in vivo* controls (**Figure 1A**). A total of 39 mice (corresponding to 192 *in vitro* fragments and 30 age-matched testis) were used in the current study. A flow chart of the study is available (**Figures 1B, C**).

## Materials and methods

### Ethical approval and housing

The animal care and use committee of Rouen University (N/23-11-12/46/11-15) approved all experimental procedures performed in the study. CD-1^®^ IGS male mice (*Mus musculus*; Charles River Laboratories, L’Arbresle, France) were housed under a 12:12 hr light/dark cycle with controlled room temperature (23°C ± 3°C) and humidity (50% ± 20%). Water and food was provided *ad libitum*.

### Mice testicular tissue collection

To ensure that the seminiferous tubules contained no germ cells more advanced than gonocytes or spermatogonia, testes were obtained from 6.5-day-old CD-1 male mice. Indeed, from 3.5 to 5.5 d*pp*, gonocytes migrate from the centre to the peripheral region of the seminiferous tubules to become spermatogonia (23). Therefore, at 6.5 d*pp*, testicular tissue is made up of spermatogonia – identified with their localization adjacent of the basement membrane of the seminiferous tubules –. The tunica albuginea was immediately removed in a phenol red-free minimum essential medium alpha (α-MEM; 41061-029; Gibco^®^ by Thermo Fisher Scientific Inc., Waltham, Massachusetts, USA) maintained at 4°C.

### Controlled slow frozen cryopreservation

Testicular tissue cryopreservation was performed according to the protocols previously developed by our team for CSF (24). Briefly, the freezing media consisted of Leibovitz-L15 medium (L15; L5520; Sigma-Aldrich^®^, Saint-Quentin Fallavier, France) supplemented with 1.5 M of dimethylsulfoxide (DMSO; D8418; Sigma-Aldrich^®^), 0.05 M of sucrose (S1888; Sigma-Aldrich^®^), 3.4 mM of (±)-α-Tocopherol (vitamin E; T3251; Sigma-Aldrich^®^), and 10% (v/v) foetal calf serum (FCS; CVFSVF000U; Eurobio AbCys, Courtaboeuf, France). The testes were placed in a CryoTube^®^ containing 1.3 ml of cryoprotective medium and were equilibrated for 30 min at 4°C. Samples were frozen with a programmable freezer (Freezal^®^, Air Liquide^™^, Marne-la-Vallée, France): start at 5°C, -2°C/min to -9°C, temperature stabilization for 7 min, -0.3°C/min to -40°C, and finally -25°C/min to -150°C. Then, the samples were stored in liquid nitrogen.

Frozen testicular tissues were thawed 1 min at RT, during 3 min by incubation at 37°C in a thermostatic water bath and then successively incubated into four baths of thawing solutions with decreasing cryoprotective agent concentrations for 5 min at 4°C at each step: (i) 1.0 M DMSO, 0.05 M sucrose, 10% FCS; (ii) 0.5 M DMSO, 0.05 M sucrose, 10% FCS, (iii) 0.05 M sucrose, 10% FCS and (iv) L15, 10% FCS without cryoprotective agent.

### Testicular tissue culture and handling

Testicular explants were cultured according to the previously published *in vitro* culture technique (17). Because the robustness of a transcriptomic analysis will be enhanced if tissue samples are stabilized as soon as possible after excision, a maximum time interval of 20 min from manipulation (*in vivo* excision or *in vitro* removal of the central necrotic part area) to preservation (*e*.*g*., equilibration at 4°C before CSF) has been informally adopted to maintain the integrity of tissue components. Briefly, testicular fragments were cultured in 6-well plate (130184; Thermo Fisher Scientific Inc.). The culture media used for this study was a medium composed of α-MEM plus 10% (v/v) KnockOut™ serum replacement (KSR) (10828-010; Gibco^®^), 5 µg/mL gentamicin (G1397; Sigma-Aldrich^®^), and retinol (all-*trans* retinol; R7632; Sigma-Aldrich^®^) at a concentration of 10^-6^ M. An alternate supplementation of retinol (Rol) was performed every 8 days to mimic the physiological timing of entry into differentiation of spermatogonia. At D4, D16, and D30, testicular explants were pooled – after removal of the central necrotic part of the tissue representing about half of the mass of the explants – from 12 testicular tissue explants per biological replicate for the RNA-Seq study. Remaining testicular explants were used for evaluation of morphological and immunofluorescence evaluations. Testicular tissues from 6.5 d*pp* (fresh and cryopreserved), 8.5 d*pp*, 22.5 d*pp*, and 36.5 d*pp*, tissues were used at *in vivo* control counterparts.

### Histological and immunofluorescence analyses

#### Tissue fixation and processing

Tissues were fixed at RT, in Bouin’s solution (HT10132; Sigma-Aldrich^®^) and paraformaldéhyde (PFA, P6148; Sigma-Aldrich^®^) at 4% for 2 hr (explants) and overnight (testis). Then, tissues were dehydrated in a graded series of ethanol in the Citadel 2000 tissue processor (12612613, Thermo Fisher Scientific Inc.) and embedded in paraffin. Sections (3 µm thick) were cut using a microtome (JungRM 2035; Leica Microsystems^©^ GmbH, Wetzlar, Germany). Three serial tissue sections were mounted on each Polysine^®^ slide (J2800AMNZ; Thermo Fisher Scientific Inc.) who were coded for future blinded analysis. For each repetition of each experiment, two to three slides separated by a minimum interval of 45 µm were examined and quantify to obtain a more accurate and global assessment of the tissue. Evaluations were assessed on 30 cross-sectioned tubules per slide if possible.

#### Immunofluorescence stainings

All the antibodies used in this study for immunofluorescence techniques are presented in **Table S1** and washes were performed with phosphate buffer saline containing Tween (PBST). First, to assess the entry into meiosis initiation of intratubular cells, we carried out immunofluorescences with the stimulated by retinoic acid gene 8 (STRA8) protein. To unmask antigenic sites, tissue sections fixed in PFA4% were incubated in citrate buffer (T0050, Diapath) at 96°C for 40 min and cooled for 20 min at RT. To avoid nonspecific staining, sections were blocked with a solution of 5% bovine serum albumin (BSA) plus 5% horse serum (HS) for 30 min. Slides were then incubated for 90 min at RT with primary antibody. Then, sections were incubated with secondary antibody coupled to anti-rabbit biotin-conjugated. Finally, sections were incubated with an Alexa Fluor^®^ 594-conjugated streptavidin for 30 min at RT. Sections were rinsed, dehydrated with ethanol and mounted in Vectashield (H-1000, Vector laboratories, Burlingame, CA, USA) with Hoechst 33342 (B2261; Thermo Fisher Scientific Inc). Negative controls were performed with mouse or rabbit immunoglobulin G (IgG).

#### HES colorations and morphological evaluations

For testicular explants and corresponding controls fixed with Bouin’s solution, slides were stained with Hemalun eosin saffron (HES) to have an appreciation of testicular morphology and to appreciate the distinction of the different stages of the germ cells present within the seminiferous tubules over time. We assessed the average surface area of the seminiferous tubules, the number of cells per seminiferous tubule, the intratubular cell density, and the progression of spermatogenesis throughout key time points of the first spermatogenic wave. Images were observed under a light microscope (DM4000B^®^; Leica Microsystem^©^ GmbH) equipped with Leica Application Suite^®^ software (LAS^®^; Leica Microsystem^©^ GmbH).

### RNA extraction, library preparation and sequencing

#### RNA extraction

Total RNA was extracted from testicular samples using RNeasy Micro kit (74004; Qiagen, Courtabœuf, France) according to the manufacturer’s instructions and stored at -20°C until use. For *in vitro* cultured testicular explants, the central necrotic area (inherent of long-term culture system) was carefully removed before RNA extraction. To avoid contamination with genomic DNA, extracted RNA was incubated with two units of TURBO DNA-free™ kit (AM1907; Life Technologies™, Carlsbad, California, USA) for 45 min at 37°C. The amount and purity of the RNA samples were measured with a NanoDrop™ spectrophotometer (ND2000; NanoDrop™ Technologies, Wilmington, DE, USA). Only samples with A260:A230 ratio values ≥1.80 were chosen for further analysis. The RNA integrity was also analyzed by capillary gel electrophoresis on RNA 6000 Nano kit chips (5067-1511; Agilent Technologies, Courtabœuf, France) with the Agilent Bioanalyzer 2100 system (G2939BA; Agilent Technologies). Only those samples with an RNA Integrity Number (RIN) value ≥8 were chosen for further analysis. Total RNA extracted material were stocked in liquid nitrogen (LN_2_).

#### Library preparation

RNA-Seq libraries were generated from 800 ng of total RNA using TruSeq Stranded mRNA LT Sample Preparation Kit (20020595; Illumina^®^, San Diego, California, USA), according to manufacturer’s instructions. Briefly, following purification with poly(T)-oligo attached magnetic beads, the mRNA was fragmented using divalent cations at 94°C for 2 min. The cleaved RNA fragments were copied into first strand cDNA using reverse transcriptase and random primers. Strand specificity was achieved by replacing dTTP with dUTP during second strand cDNA synthesis using DNA Polymerase I and RNase H. Following addition of a single ‘A’ base and subsequent ligation of the adapter on double stranded cDNA fragments, the products were purified and enriched with PCR (30 sec at 98°C; [10 sec at 98°C, 30 sec at 60°C, 30 sec at 72°C] ×12 cycles; 5 min at 72°C) to create the cDNA library. Surplus PCR primers were further removed by purification using AMPure XP beads (A63881; Beckman-Coulter, Villepinte, France) and the final cDNA libraries were checked for quality and quantified using capillary electrophoresis.

#### RNA-Sequencing

The prepared RNA samples were quantified on a Qubit™ Fluorometer (Q33226; Thermo Fisher Scientific Inc.) and adjusted to 800 ng. *Mus musculus* organism was chosen with mm10 as genome assembly. The library preparation used was SQ00/SIL-09-PE (for Stranded mRNA-Seq/standard quantity) with read length of SQ00/HS-1×50_40 (with 1×50 bases and an adapter size of 120 bp) and saved in .fastq data files. In total, 36 samples were run on an Illumina^®^ HiSeq 4000 Sequencing technology (SY-401-4001; Illumina^®^) for single-read 50 bp in Poly(A)-enriched mode. Image analysis and base calling were carried out using RTA v.2.7.7 and bcl2fastq v.2.17.1.14. The mRNA-Seq data are deposited at Sequence Read Archive (NCBI)^1^.

### Data preprocessing and exploration

#### Read mapping

Reads from each individual sample were aligned to the mm10 release of the mouse genome with STAR (version 2.5.2a) (25), using previously published approaches (26–30). Briefly, the STAR program was first run for each fastq file, using the RefSeq transcript annotation (GTF format) of the mouse genome (release mm10). Exon junction outputs were then added to a splice junction set. STAR was next run a second time, complemented by the splice junction dataset, to produce a final alignment file (BAM format) for each sample.

#### Transcriptome quantification

RefSeq transcripts were quantified with StringTie (version 1.3.3), with default settings applied (31). Transcript abundances were next normalized with Ballgown (available in the StringTie suite), expressed as reads per kilobase of exon model per million (RPKM).

#### Statistical filtration and clustering analysis

The statistical filtration of the genes showing a differential expression across experimental samples was performed using the Annotation, Mapping, Expression and Network (AMEN) suite of tools (32). We first selected 12,565 genes “detectable” or “expressed” genes, defined as those for which abundance levels exceeded 0.5 RPKM in at least one experimental condition (median value of sample duplicates). Next, we compared *in vitro vs*. *in vivo* (*i*.*e*., D4 *vs*. 10.5 d*pp*; D16 *vs*. 22.5 d*pp*; and D30 *vs*. 36.5 d*pp*) and fresh *vs*. frozen (6.5 d*pp* CSF *vs*. 6.5 d*pp* and D30 CSF *vs*. D30) testicular tissue samples and selected genes yielding at least one-fold change greater than or equal to 2.0 (median values of sample duplicates). A linear models for microarray data (LIMMA) statistical test was used to identify genes with significant abundance variations across samples (F-value adjusted with the FDR method: *P*<0.05) (33). The differentially expressed genes (DEGs) were clustered into eight (C1-C8) and five (F1-F8*) expression patterns with the unsupervised HCPC algorithm for *in vitro vs*. *in vivo* and fresh *vs*. frozen, respectively (34).

#### Functional profiling

The *g:GOSt* function was used to known functional information sources and detects statistically significantly enriched terms using *g:SCS* threshold fixed at 0.05. In order to consider only the elements that emerge from the batch for *in vitro vs*. *in vivo*, DEGs selected for this analysis have a p_adj_<6×10^-8^. The g:GOSt database versions used in the present study are Ensembl 104 and Ensembl Genomes 51^2^. In addition to GO Terms with the use of biological process (BP), cellular component (CC), and molecular function (MF)^3^, the g:GOSt includes pathways from Reactome (REAC)^4^, and WikiPathways (WP)^5^ up-to-date data sources.

### Differential expression bioinformatic analysis

#### Bioinformatics analysis

Reads were aligned against the reference genome (GRCm38) using STAR (2.7.3a; 25) with default parameters. Gene expression was then calculated with HTseq (0.12.4; 35) with *–mode intersection-nonempty*.

#### Principal component analysis and sample distance map

The raw count data from all samples were transformed with the varianceStabilizingTransformation (*vst*) function from DESeq2. We then calculated the Euclidean distance between samples on the transformed counts and the samples were then clustered using the R function *hclust*. We also performed a PCA on the vst transformed counts using the 500 most variable genes. The biological samples who did not meet the quality checks were therefore eliminated from this bioinformatic analysis.

#### Differential analysis fold-chance

Differential analysis was then performed with R, using DESeq2^6^. Each condition was compared to another, and raw p-values were adjusted by the method of Benjamini and Hochberg to control a false discovery rate (FDR) (36). DEGs selected for Volcano Plots analysis have a |Log_2_ fold change| greater or equal to 2.0 and a |Log_10_ p_adj_| greater or equal to 3.0.

#### KEGGs pathway

For KEGGs pathways evaluation, we considered genes with both a log2 fold-change of at least 1.5 and adjusted p-values under 0.001 as differentially expressed here. The *g:GOSt* function^7^ from R package *g:Profiler* was used to detect statistically significantly enriched terms.

### Packages and statistical analyses

Packages were implemented in the R statistical environment Team (2011 – R Foundation for Statistical Computing, Vienna, Austria. URL^8^). Other statistical analyses were performed with GraphPad Prism version 8.2.1 (279) (GraphPad Software, La Jolla, California). The Mann-Whitney test was used for unpaired rank comparisons and the nonparametric Wilcoxon test was used for paired rank comparisons determined by a unilateral one-tailed. The one-way ANOVA test was used for the evaluation of the assay’s reproducibility, followed by a Tukey’s multiple comparison *post-hoc* test. Histological and morphological data are presented as the means ± s.e.m. and a p-value *P*<0.05 was considered to be significant.

## Results

### Characterization of differentially expressed genes

#### *In vitro* culture-induced modifications in the testicular transcriptome

**Figure 2:**
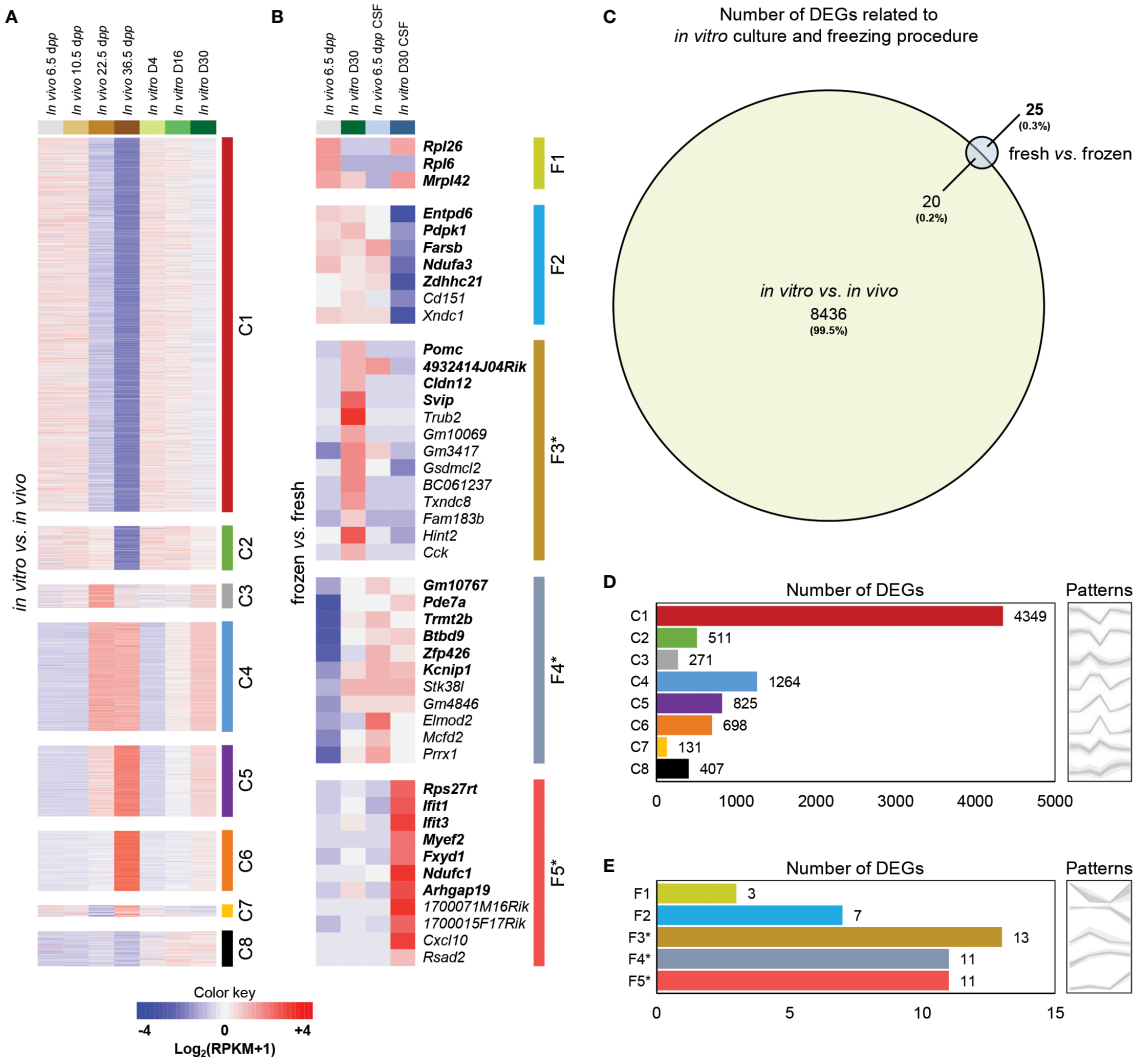
Global gene expression profiling of testicular tissue samples in response to *in vitro* culture or freezing procedures. False-color heatmap summarizing the patterns defining the global concentrations for transcripts across the entire sample set focused on the impact of *in vitro* culture **(A)** and freezing procedure **(B)**. Each row corresponds to the mean of biological replicate samples: *in vivo* 6.5 (fresh: n=3; CSF: n=2), 10.5 (n=3), 22.5 (n=3), 36.5 (n=2) d*pp*; *in vitro* D4 (n=3), D16 (n=2), and D30 (fresh tissue: n=3; CSF: n=1). C1 to C8 and F1 to F5* refer to the individual patterns for the impact of *in vitro* culture and freezing procedure, respectively. DEGs only present for fresh *vs*. frozen are in bold. Fold change values are shown with a red or blue background indicating up-regulation or down-regulation, respectively. A color key-scale is shown for standardized values in Log_2_(RPKM+1). **(C)** Area-proportional Venn Diagram showing the overlap of significantly DEGs in *in vitro vs*. *in vivo* (with 8436 uniquely mapped transcripts from a total of 8456 DEGs) compared to fresh *vs*. frozen (with only 25 uniquely mapped transcripts from a total of 45 DEGs). Numbers labeled in each part show the amount of the genes with the corresponding expression patterns. The number of significantly DEGs included in each pattern are shown for the impact of *in vitro* culture **(D)** and freezing procedure **(E)**. Grey patterns are the graphical visualization of the average expression levels of the several patterns (with the variations between repetitions). F3* (23.1%), F4* (18.2%), and F5* (18.2%) were patterns with a large proportion of DEGs starting with ‘Gm-’ or ending with ‘Rik’ who are predicted genes or annotated genes that do not have a canonical name (yet). Based on Ensembl biotype classifications in mouse (https://www.ensembl.org/Mus_musculus/Info/Index), genes starting with ‘Gm-’ or ending with ‘Rik’ include protein-coding RNAs, long non-coding RNAs, and antisense transcripts. Interestingly, genes with the ‘Gm’ prefix are enriched for pseudogenes. CSF, controlled slow freezing; DEGs, differentially expressed genes; d*pp*, days *postpartum*; RPKM, reads per kilobase million.

An initial analysis was performed to identify patterns with corresponding DEGs. Then, an analysis of pattern’s Gene Ontology (GO) Terms was carried out. Finally, an investigation of known testicular cell genes was settled to characterize biological samples and determine the potential impact of (i) *in vitro* culture and (ii) freezing procedure. Considering that *in vitro* culture does not allow achieving a spermatic yield similar to the physiological conditions observed *in vivo*, we wanted to examine the biological and molecular changes that differ throughout the first wave of spermatogenesis. To this end, we compared global gene expression between cultured testicular explants and physiological corresponding testicular tissue by RNA-Seq. A clustering analysis on the resulting set of genes allowed us to highlight eight expression patterns (termed C1-8) for the impact of *in vitro* culture on the first spermatogenic wave (**Figures 2A, D**). A total of about 8,456 DEGs were shown between cultured testicular explants and *in vivo* controls (especially for genes related to advanced stages of spermatogenesis) (**Figure 2C**). C1-C2, C3-C7, and C8 can be regrouped due to their broad expression profiles: cluster C1-C2 corresponds to 4,860 genes transiently expressed in the testis during its maturation and predominantly down-regulated in cultured tissues; cluster C3-C7 corresponds to 3,189 genes poorly expressed during the maturation process but up-regulated in cultured tissues, and cluster C8 corresponding to 407 genes highly expressed only at the end of *in vitro* culture.

In order to investigate the molecular pathways involved in the alterations of the maturation process throughout *in vitro* culture, we performed a functional analysis based on a generic GO Terms enrichment on the expression patterns (**Figure S1A**). The patterns C1 (251 GO Terms) and C2 (107 GO Terms) were mainly related in general mechanisms such as cytoplasm, intracellular cell part and organelle, nucleus, metabolic biological processes, and protein binding. Interestingly, the pattern C3 (39 GO Terms) was linked to meiotic cell cycle, chromosome segregation, and nuclear division. A strong up-regulation of genes associated with this pattern can be observed at 22.5 d*pp* (**Figures 2A, D**), a timing during which pachytene spermatocytes I perform their strict pairing of homologous chromosomes and when recombination form allows crossing-over. The pattern C4 (26 GO Terms) was associated with cilium, sperm motility, microtubule activity, and CatSper complex. These Terms are strongly up-regulated at 22.5 and 36.5 d*pp*, representing the initiation of gene expression necessary for spermiogenesis (a late stage of spermatogenesis that remains highly impaired in culture). As expected, the pattern C5 (10 GO Terms) corresponding to the majority cell population of elongated flagellated spermatids at 36.5 d*pp* was related to sperm part, spermatogenesis, acrosome vesicle, and reproduction. The pattern C7 (4 GO Terms) was linked to cytoplasmic part and ion binding. The pattern C8 (102 GO Terms) was associated to response to stress, inflammatory response, immune system process, and cytokine production. Mostly up-regulated for D16 and D30, these transcriptional data suggest an important inflammatory response of the testicular explants during *in vitro* culture.

#### Minimal impact of freezing in the testicular transcriptome

Five patterns were highlighted for the impact of freezing procedure (**Figures 2B, E**). A total of only 25 specific genes were considered as differentially expressed between cryopreserved and fresh tissues without the impact of *in vitro* culture (**Figure 2C**): Cluster F1 (*Rpl26*, *Rpl6*, *Mrpl42*), Cluster F2 (*Entpd6*, *Pdpk1*, *Farsb*, *Ndufa3*, *Zdhhc21*), Cluster F3* (*Pomc*, *4932414J04Rik*, *Cldn12*, *Svip*), Cluster F4* (*Gm10767*, *Pde7a*, *Trmt2b*, *Btbd9*, *Zfp426*, *Kcnip1*), Cluster F5* (*Rps27rt*, *Ifit1*, *Ifit3*, *Myef2*, *Fxyd1*, *Ndufc1*, *Arhgap19*). This functional enrichment analysis revealed a high number of overlapping genes (20 DEGs; 44,5%) between freezing procedure and *in vitro* culture impact. These observations suggest a minor impact of the freezing protocol used to cryopreserve prepubertal testicular tissues.

For the impact of freezing procedure, the patterns F1 (6 GO Terms), F3* (1 GO Terms), and F5* (3 GO Terms) were associated to ribosome, regulation of appetite, and response to interferon/defense, respectively (**Figure S1B**). The interpretation of these data is relatively complex due to the low number of GO Terms per pattern. However, although freezing has very limited impact on testicular tissue, we observe a weak response of frozen and cultured testicular tissue through a delicate persistent response to external aggression.

### Meiotic progression and differentiation of germ cells

#### Meiosis initiation and germ cell differentiation

**Figure 3:**
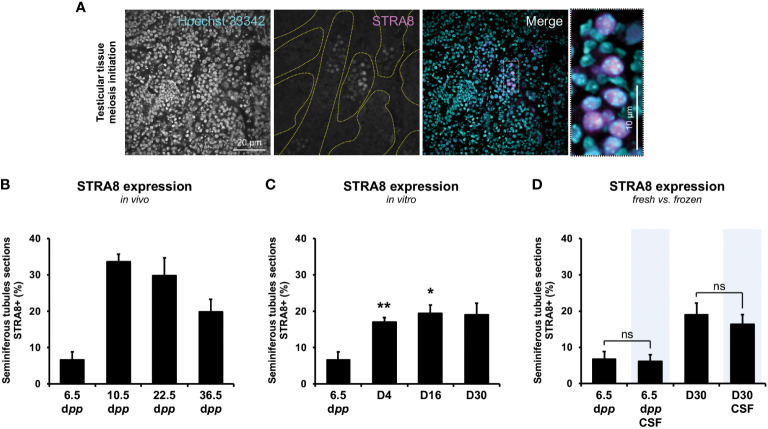
Evaluation of the onset of meiosis initiation throughout the first spermatogenic wave. The presence of STRA8 **(A)**, a protein enabling the initiation of the transition between mitosis and meiosis, was assessed throughout the first spermatogenic wave for *in vivo* testicular tissues **(B)** and *in vitro* testicular explants obtained throughout 30-day-organ culture **(C)** (n=3×6 fragments per condition ×3 slides ×30 seminiferous tubules) at D4, D16, and D30. Representative data of STRA8 staining is presented. A comparison between fresh and frozen prepubertal testicular explants was assessed at the beginning (6.5 d*pp*) and the end (D30 and D30 CSF) of the culture **(D)**. Testes from mice aged of 6.5, 10.5, 22.5, and 36.5 d*pp* were used as age-matched corresponding *in vivo* control (n=3 testis ×3 slides ×30 seminiferous tubules). Seminiferous tubules were delimited with dotted yellow lines. Magnification ×200 (with additional ×5 numerical zoom for micrographs); scale bar: 20 μm (or 10 μm for zoomed micrographs). Kruskal-Wallis tests followed with Dunn’s post-test were applied for **(A–C)** and Mann-Whitney tests was applied for statistical analyses. Data are expressed as mean ± s.e.m. *P<0.05; **P<0.01. CSF, controlled slow freezing; d*pp*, days *postpartum*; ns, not significant; STRA8, stimulated by retinoic acid gene 8.

The presence of the onset of meiotic prophase retinoic acid protein STRA8 into seminiferous tubules follows the same dynamics *in vitro* as *in vivo*. However, a lower proportion of STRA8-expressing seminiferous tubules is observed in culture at D4 (17.2 ± 5.28) and D16 (19.5 ± 2.21) compared to 10.5 (33.6 ± 2.06; *P*<0.001) and 22.5 d*pp* (19.5 ± 4.95; *P*<0.01) (**Figures 3B, C** **)**. Freezing procedure does not prevent entry into meiosis of spermatogonia before or after *in vitro* culture (*P*≥0.05) (**Figure 3D**).

#### Morphological analysis and germ cell transcripts

**Figure 4:**
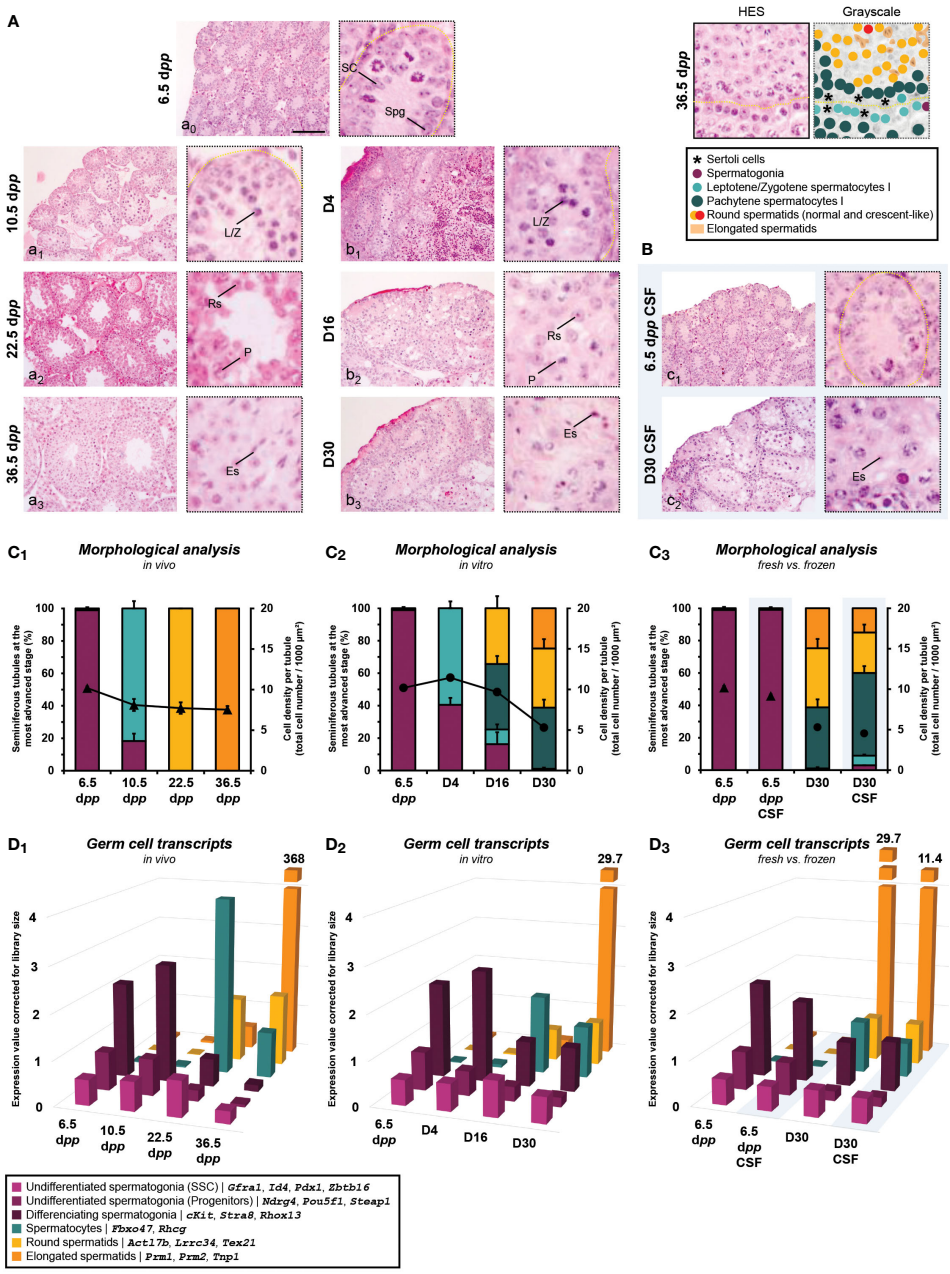
Testicular tissue assessment during the first wave of spermatogenesis. Histological analysis performed in HES of *postpartum in vivo* testicular tissues **(A, a_0_-_3_)** (n=3 testis ×3 slides ×30 seminiferous tubules) and testicular explants obtained throughout *in vitro* 30-day-organ culture of fresh **(A, b_1_-_3_)** or frozen **(**by CSF; **B, c_1_-_2_)** tissues (n=3×6 fragments per condition ×3 slides ×30 seminiferous tubules). Magnification ×200 (with additional ×5 numerical zoom for micrographs); scale bar: 100 μm. The percentage of seminiferous tubules containing differentiated germ cells at the most advanced stage and cell density per tubule (▲, *in vivo*; ●, *in vitro*) were evaluated for *in vivo* **(C_1_)**, *in vitro* **(C_2_)**, and fresh *vs*. frozen **(C_3_)**. Data are presented as mean ± s.e.m. RNA-Seq data of known genes corresponding to specific germ cell stages were presented to have a relative comparison of different germ cell types between several conditions within *in vivo* testicular tissues **(D_1_)**, *in vitro* explants **(D_2_)** and fresh *vs*. frozen **(D_3_)**. Data are presented as mean expression values corrected to library size. CSF, controlled slow freezing; d*pp*, days *postpartum*; Es, elongated spermatid; HES, hemalun eosin saffron; L/Z, lepto-zygotene spermatocyte I; P, pachytene spermatocyte I; Rs, round spermatid; SC, Sertoli cell; Spg, spermatogonia.

Regarding the kinetics of the first wave of spermatogenesis, there is a delay at D4 in the timing of entry of germ cells into meiosis with a lower percentage of seminiferous tubules containing Leptotene/Zygotene spermatocyte I cells (59.5 ± 4.24%) in comparison to 10.5 d*pp* (81.7 ± 4.54%; *P*<0.0001) (**Figures 4A** and **C_1,2_**). A lower number of seminiferous tubules were observed with haploid cells *in vitro* at D16 (34.4 ± 7.43% for round spermatids) and D30 (36.5 ± 5.71% for round spermatids and 24.7 ± 4.69% for elongated spermatids) than *in vivo* at 22.5 (8.10 ± 0.74% for round spermatids; *P*<0.0001) and 36.5 d*pp* (100 ± 0.00% for round spermatids and elongated spermatids; *P*<0.0001), respectively (**Figures 4A** and **C_1,2_**). In addition, we observed an increase in intratubular cell density at D4 for the tissue cultured *in vitro* (11.4 ± 0.14 cells/1000 µm²) compared to *in vivo* at 10.5 d*pp* (8.10 ± 0.74 cells/1000 µm²; *P*<0.0001) (**Figures 4A** and **C_1,2_**). This observation is due to the fact that testicular explants have a lower seminiferous tubule expansion as well as a lower entry into meiosis, resulting in a higher cell number per µm². This observation is counterbalanced with a strong reduction in cell density at D30 for tissue grown *in vitro* (5.29 ± 0.20 cells/1000 µm²) compared to *in vivo* at 36.5 d*pp* (7.53 ± 0.44 cells/1000 µm²; *P*<0.01) (**Figures 4_C1-2_**). These observations could be explained by a lower proliferation of germ cells in culture while suggesting a well-known mechanism of disappearance by autophagy of maturing cells that have difficulty passing the pachytene checkpoint.

Regarding the impact of freezing procedure, a lower number of seminiferous tubules were observed with haploid cells at D30 CSF (25.0 ± 4.89% for round spermatids) than at D30 (36.5 ± 5.71% for round spermatids; *P*<0.05) (**Figures 4A, B** and **C_3_**). Regarding the cell density obtained after culture of frozen or fresh tissue, no difference could be observed (*P*≥0.05*)* (**Figures 4A, B** and **C_3_**). These observations suggest that testicular tissue freezing does not have a major impact on the first wave of spermatogenesis initiation and development.

Although the proportions of germ cells transcripts were globally comparable between the *in vitro* cultures and the age-matched controls *in vivo*, we observed a small decrease in spermatocyte-related transcripts observed at D16 (1.77) compared to 22.5 d*pp* (4.00) (**Figures 4D_1-2_**). In addition, the levels of protamines (*Prm1*, *Prm2*) and spermatid nuclear transition protein 1 (*Tnp1*) expressions drop at the end of the *in vitro* cultured condition (29.7) (**Figures 4D_2_**) and even more from frozen tissue (11.4) (**Figures 4D_3_**) compared to *in vivo* age-matched controls (368) (**Figures 4D_1_**). This observation highlights the low yield of spermatogenesis obtained under non-physiological conditions.

#### Expression value of testicular cells corrected for library size

In our experimental setup, the RNA of whole testis or pool of testicular explants were used for sequencing. Because the study of the testis is not limited to germ cells, we studied transcripts derived from somatic testicular cell. In addition to germ cells transcripts (*Ddx4*, *Prm1*, *Prm2*) and in order to understand the contribution of somatic cells in the gene expression differences, we analyzed the expression of Sertoli (*Vim*, *Gata4*, *Ar*, *Amh*, *Sox9*), Leydig (*Cyp17a1*, *Cyp11a1*, *Hsd3b1*, *Ccn5*), and peritubular and blood vessel transcripts (*Bgn*, *Dcn*, *Acta2*) cells genes.

**Figure 5:**
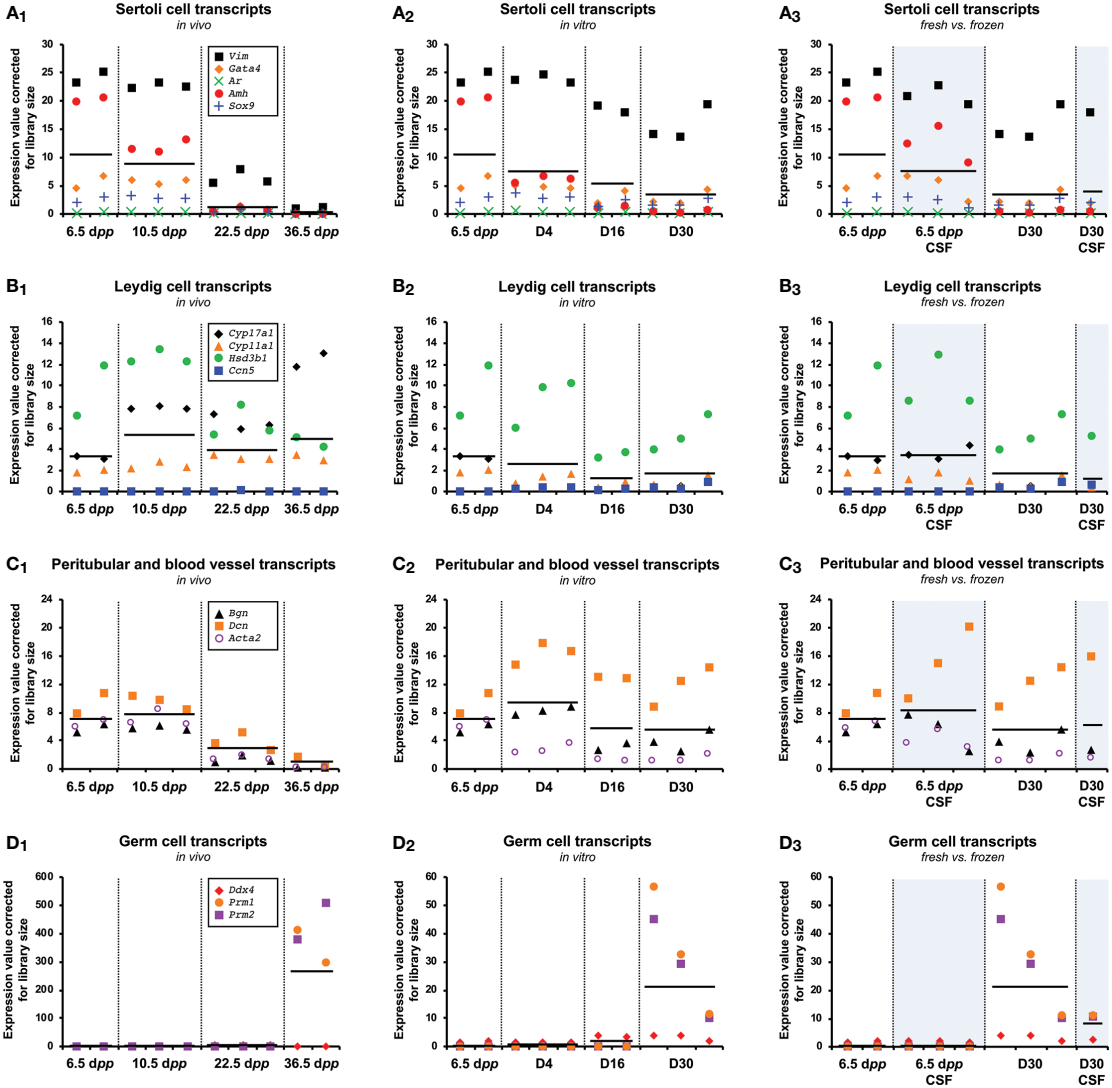
Cell gene expression during the first spermatogenic wave. Known Sertoli (*Vim*, *Gata4*, *Ar*, *Amh*, *Sox9*) **(A_1-3_)**, Leydig (*Cyp17a1*, *Cyp11a1*, *Hsd3b1*, *Ccn5*) **(B_1-3_)**, peritubular/blood vessel (*Bgn*, *Dcn*, *Acta2*) **(C_1-3_)**, and germ cell (*Ddx4)*/elongated spermatids (*Prm1*, *Prm2*) **(D_1-3_)** markers expressed in the testis or testicular explants during the first wave of spermatogenesis for *in vivo* **(A_1_, B_1_, C_1_, D_1_)**, *in vitro* **(A_2_, B_2_, C_2_, D_2_)** and fresh *vs*. frozen **(A_3_, B_3_, C_3_, D_3_)** samples. Horizontal black lines indicate the mean expression values in each group. CSF, controlled slow freezing; d*pp*, days *postpartum*.

For *in vivo*, the expression of genes related to Sertoli cells (**Figure 5A_1_**) and peritubular and blood vessel transcripts (**Figure 5C_1_**) decreased during the first wave of spermatogenesis, possibly reflecting the increase in germ cell population, especially at 36.5 d*pp* with a large presence of spermatids (**Figure 5D_1_**). The analysis of the expression of Leydig cells specific genes show no global difference across the first wave, although we observed an increase of the expression of Cytochrome P450 gene *Cyp17a1* at 36.5 d*pp* (**Figure 5B_1_**). For *in vitro* culture, equivalent profiles were observed with a lower cell-dilution effect (**Figures 5A_2_, B_2_**, and **C_2_**) due to a lower spermatic yield. However, *Cyp17a1* expression did not increase at D4 and throughout the culture while the Leydig cell marker *Hsd3b1* is still present at levels comparable to those *in vivo*, suggesting a defective maturation of the Leydig cells *in vitro*. Besides, despite the fact that we pooled 12 testicular tissue explants per biological replicate for *in vitro* conditions, we observed a rather important variability in the expression of genes related to highly differentiated germ cells (*Prm1*, *Prm2*) (**Figure 5D_2_**). Concerning the comparison between fresh and frozen tissue, the expression levels of the studied genes are not different from a somatic cell point of view (**Figures 5A_3_, B_3_**, and **C_3_**). Regarding germ cells, a lower yield of spermatogenesis is observed (**Figure D_3_**).

### Characterization of the Relationship between *in vitro* and *in vivo*

**Figure 6:**
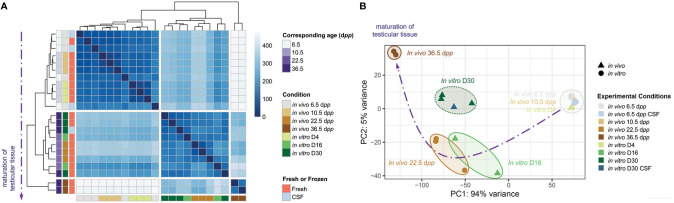
Assessment of the maturation of testicular tissue at key stages of the first spermatogenic wave. **(A)** Correlation map representing all biological samples conditions and repetitions. Each row corresponds to the mean of biological replicate samples: *in vivo* 6.5 (fresh: n=3; CSF: n=2), 10.5 (n=3), 22.5 (n=3), 36.5 (n=2) d*pp*; *in vitro* D4 (n=3), D16 (n=2), and D30 (fresh tissue: n=3; CSF: n=1). A kinetic evolution curve of the transcriptome of maturing testicular tissues is represented in dotted purple arrow. **(B)** Principal component analysis (PCA). Representation of the clusters on the map of the gene expression dataset are plotted by the first two principal components (PC) PC1 (94% variance) and PC2 (5% variance). The position of individual samples is indicated in the PCA individual factor map by colored spots which indicate *in vivo* testicular tissues controls (▲) and *in vitro* explants (●) cultured from fresh or frozen tissues. Five separate clusters of samples are represented with ellipse delimitations. CSF, controlled slow freezing; d*pp*, days *postpartum*.

We observed a successful maturation *in vitro* of the testicular tissue with respect to the physiological kinetics of testicular maturation (**Figure 6A**). Explants matured until D4 and D10 gathered with 6.5 and 10.5 d*pp* age-matched *in vivo* controls. In addition, cultures from D16 gathered with 22.5 d*pp*. However, we noticed that testicular tissues at D30 do not cluster with their corresponding 36 d*pp* age-matched *in vivo* controls and are grouped with the D16 (and the 22.5 d*pp*). Therefore, we observed an overall expression profile of tissues cultured to D30 comparable to that of a D16, suggesting an abnormal kinetic in maturation between D16 and D30. For explants grown from frozen or fresh tissue, no difference was observed, suggesting once again that freezing have no harmful impact on the fate of the cryopreserve testicular tissue.

We performed a principal component analysis (PCA) for the transcriptome data and draw a 2-dimensional plot for principle component (PC) 1 (94% variance) and PC2 (5% variance) (**Figure 6B**). As well, the kinetics of the first wave of spermatogenesis is respected *in vitro*. Indeed, the different times corresponding to the advancement of spermatogenesis are grouped in the form of clusters. PC1 and PC2 scores of *in vitro* D4 samples were similar to those of corresponding *in vivo* 10.5 d*pp* testis samples. While PC1 scores are similar between *in vitro* D16 and corresponding 22.5 d*pp*, PC2 scores are a bit higher than *in vivo* counterparts. Regarding the timing corresponding to the end of the first wave of spermatogenesis, *in vitro* D30 samples were left behind in comparison to 36.5 d*pp* with higher PC1 and lower PC2 scores. As far as fresh or frozen conditions are concerned, they are grouped in the same clusters. However, *in vitro* culture tends to be delayed between D16 and D30 in comparison to age-matched *in vivo* controls.

Considering the fact that the freezing procedure showed only a minimal impact on gene expression and in order to have a greater power of analysis to compare the different timings of spermatogenesis, the frozen tissues were grouped with their fresh correspondents for further investigations.

### Expression kinetics of the first wave of spermatogenesis

#### Physiological first spermatogenic wave kinetic

Between 6.5 d*pp* and 10.5 d*pp*, very few differences in gene expression (only 6 DEGs) can be highlighted with a reasonable p-value and a consistent gene ratio (**Figure S2A_1_**); afterwards, almost all genes are upregulated during the first wave of spermatogenesis (**Figures S2A_2,3_** and **C_2,3_**), reporting the successful establishment of the first wave of spermatogenesis. Between 10.5 d*pp* and 22.5 d*pp*, we notice a majority of GO Terms focused on the flagellum/cilium, reproduction, and spermatozoon in formation (**Figure S2B_2_**), which is consistent with the onset of spermatogenesis. Between 22.5 d*pp* and 36.5 d*pp*, we can observe again GO Terms in connection with the flagellum but especially with nucleus organization and sperm chromatin condensation (**Figure S2B_3_**), corresponding to the establishment of spermiogenesis. As expected, between 6.5 *dpp* and 36.5 *dpp*, we observe the overexpression of genes related to the establishment of the first wave of spermatogenesis (**Figures S2C_1,3_** and **D_1,3_**). In addition, we observe the establishment of the blood testis barrier (BTB) *via* the apparition of Kyoto Encyclopedia of Genes and Genomes (KEGGs) pathways related to tight junctions, gap junctions, and adherens junctions (**Figure S3A_3_**).

#### *In vitro* first spermatogenic wave kinetic

Between 6.5 d*pp* (corresponding to D0) and D4, we can observe a response of the tissue to the cutting at the beginning of the culture (response to stimulus, wound healing) (**Figure S4B_1_**). In addition, we observe a change in the expression of genes related to GO Terms associated with vascularization (hemoglobin and blood pressure), which is consistent with the degeneration of blood capillaries after a few days of *in vitro* culture. Despite oxygen supply during organotypic gas-liquid interphase culture system, the absence of vascularization leads to a loss of physiological oxygenation (response to hypoxia and decreased oxygen level). Moreover, the direct exposition to oxygen with a constant artificial oxygenation seems to induce oxidative stress to testicular explants exposed to a more harmful environment (action on CH-OH group of donors and oxidoreductase activity). Finally, a dysregulation of steroid hormones (*e*.*g*., testosterone, steroids, and angiogenesis) is present at the beginning of the culture, which raises questions about the status of Sertoli and Leydig cells maturity and/or functionality throughout the *in vitro* culture. In addition, after only 4 days of *in vitro* culture we can observe the establishment of KEGG pathways related to steroidogenesis, hypoxy (NF-κB), and inflammation response (cytokines, chemokine, HIF-1) (**Figure S3B_1_**).

Between D4 and D16, we mainly observe the presence of DEGs linked to the establishment of spermatids and spermatozoa (GO Terms related to CatSper complex, sperm, acrosomal complex, axonemal complex, cilium, flagellum, and spermatogenesis) (**Figure S4B_2_**). Between D0 and D16, we observe the overexpression of genes related to the establishment of the first wave of spermatogenesis (**Figures S4C_1,2_** and **D_1,2_**) and the maintenance of KEGG pathways related to inflammation response between D4 and D16 (complement, natural killer cells mediated cytotoxicity, RIG-I-like receptor, IL-17) (**Figure S3B_2_**).

Even if almost all the genes are up-regulated between D16 and D30, the number of DEGs (only 58 genes) remains very small (**Figure S4A_3_**). We observe a change in the expression of genes related to GO Terms of sperm formation (sperm individualization, gamete generation, multicellular reproductive process, sperm motility) as well as DNA modifications (chromatin condensation, DNA packaging and conformation change) and immune response (innate immune response) (**Figure S4B_3_**). In addition, only eighteen genes were differencially expressed between testicular explants cultured up to D30 compared to D16 with a |Log_10_ p_adj_| greater to 3.0 (**Figure S4C_3_**), reinforcing the idea that an abnormal kinetic throughout the second half of the first spermatogenesis and/or blockage is present at the end of spermatogenesis *in vitro* with the presence of inflammatory process or immune cell recruitment. Surprisingly, only one gene is down-regulated: *Rps3a3* [log_2_-fold change=-7.13; p_adj_=9.24E-5] (**Figure S4D_3_**). Again, we can observe the maintenance of KEGG pathways related to inflammation response (IL-17, cytokine, chemokine) (**Figure S3B_3_**), symbolizing an increase in inflammation mechanisms throughout the culture.

#### Impact of the *in vitro* culture versus age-matched controls

**Figure 7:**
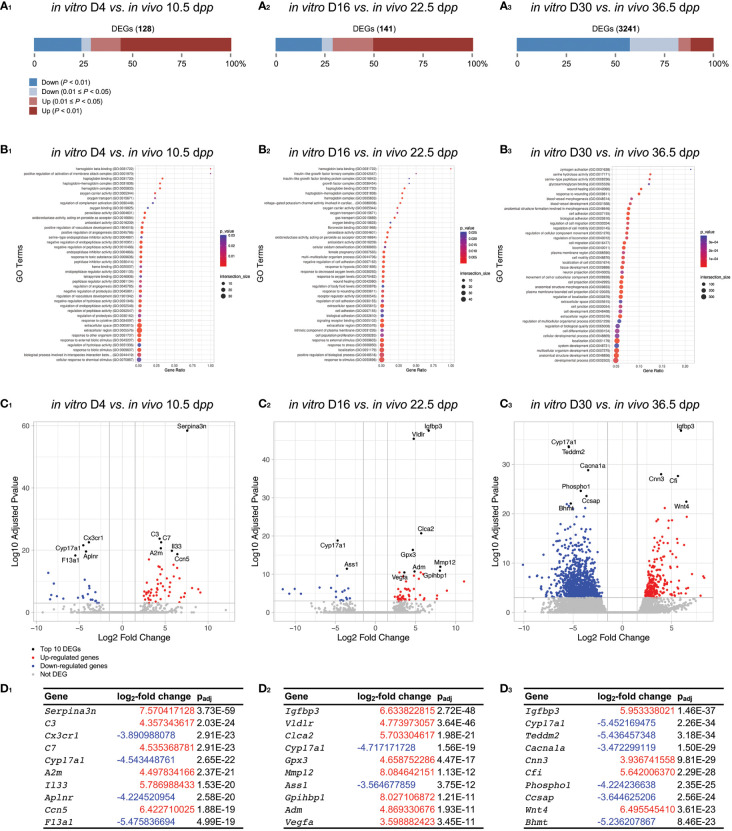
Gene-level differential expression analysis between *in vitro* and *in vivo* age-matched testicular samples. **(A)** The proportion of significantly DEGs are shown between *in vitro* and *in vivo* testicular tissue samples for D4 *vs*. 10.5 d*pp* **(A_1_)**, D16 *vs*. 22.5 d*pp* **(A_2_)**, and D30 *vs*. 36.5 d*pp* **(A_3_)**. The number of DEGs for each comparison is available in order to have an appreciation of the difference in gene expression between the two conditions compared. **(B_1-3_)** Corresponding GO enrichment dot plot. The 40 GO processes with the largest gene ratios are plotted in order of gene ratio. The size of the dots represent the number of genes in the significant DEGs associated with the GO Terms and the color of the dots represent the p_adj_ values. **(C_1-3_)** Volcano plots compare the amount of gene expression change to the significance of that change (here plotted as the log_10_ transformation of the multiple test p_adj_ value), with each point representing a single gene. The top 10 gene candidates are highlighted in black and by text labeling. The two marginal plots showing the distributions of the log_2_-fold changes and negative log10 p_adj_ values are used to show cutoff choices and trade-offs. **(D_1-3_)** Top ten DEGs with corresponding log_2_-fold change and p_adj_ value. DEGs, differentially expressed genes; d*pp*, days *postpartum*.

At D4 (**Figure 7A_1_**), we can observe mainly DEGs related to GO Terms of oxidative stress (antioxidant activity), vascularization (hemoglobin, heme, blood pressure, oxygen), and response to 4 days of culture (complement, cytokine, response to stimulus) (**Figure 7B_1_**). Those results are consistent with the degeneration of blood capillaries and the relative harmfull generated on the testicular explant at the beginning of the culture in comparison to age-matched control. Among the top-ten DEGs, we notice an over-expression of *Serpina3n*, *Complement component 3* (***C3***), *C7*, and *Il33* as well as an under-expression of *Cyp17a1* and *F13a1* (**Figures 7D_1_** and **D_3_**). In addition, we can observe the presence of KEGG pathways related to inflammation response (complement, cytokine), steroidogenesis and cell adhesion/senescence (**Figure S3C_1_**).

At D16 (**Figure 7A_2_**), we observe a dysregulation of genes linked to oxidative stress (oxygen, antioxidant activity), vascularization (hemoglobin, haptoglobin, blood pressure, oxygen transport and binding), response to hypoxia, and growth factors (insulin-like growth factor), response to stimulus/stress, wound healing, and cell adhesion (cell adhesion, biological adhesion) (**Figure 7B_2_**). Among the top-ten DEGs, we notice an over-expression of *Igfbp3*, *Vldlr*, *Gpx3*, *Mmp12*, *Vegfa* as well as an under-expression of *Cyp17a1* (**Figures 7D_2_** and **D_3_**). In addition, we can observe the maintenance of KEGG pathways related to inflammation response (complement) and hypoxia (HIF-1) (**Figure S3C_2_**).

At D30, a large proportion of genes are down-regulated when comparing to age-matched corresponding 36.5 d*pp* (**Figure 7A3**). DEGs related to GO Terms of response to cell adhesion (cell adhesion, biological adhesion), spermatozoa formation (cilium, sperm flagellum, tubulin, gamete generation), probably present due to the very low proportion of elongated spermatids *in vitro* compared to those observed physiologically at 36.5 d*pp*. In addition, GO Terms related to stress, wound healing, and tissue development (system, process, cell, *etc*.) were observed. Among the top-ten DEGs, we notice an over-expression of *Igfbp3*, *Cfi*, *Wnt4* as well as an under-expression of *Cyp17a1* (**Figures 7C_3_** and **D_3_**). Again, we can observe the maintenance of KEGG pathways related to inflammation response (complement, TNF signaling) (**Figure S3C_3_**), symbolizing a strong presence of inflammatory reaction from the beginning of the culture and up to 30 days.

### The end of the first wave of *in vitro* spermatogenesis

**Figure 8:**
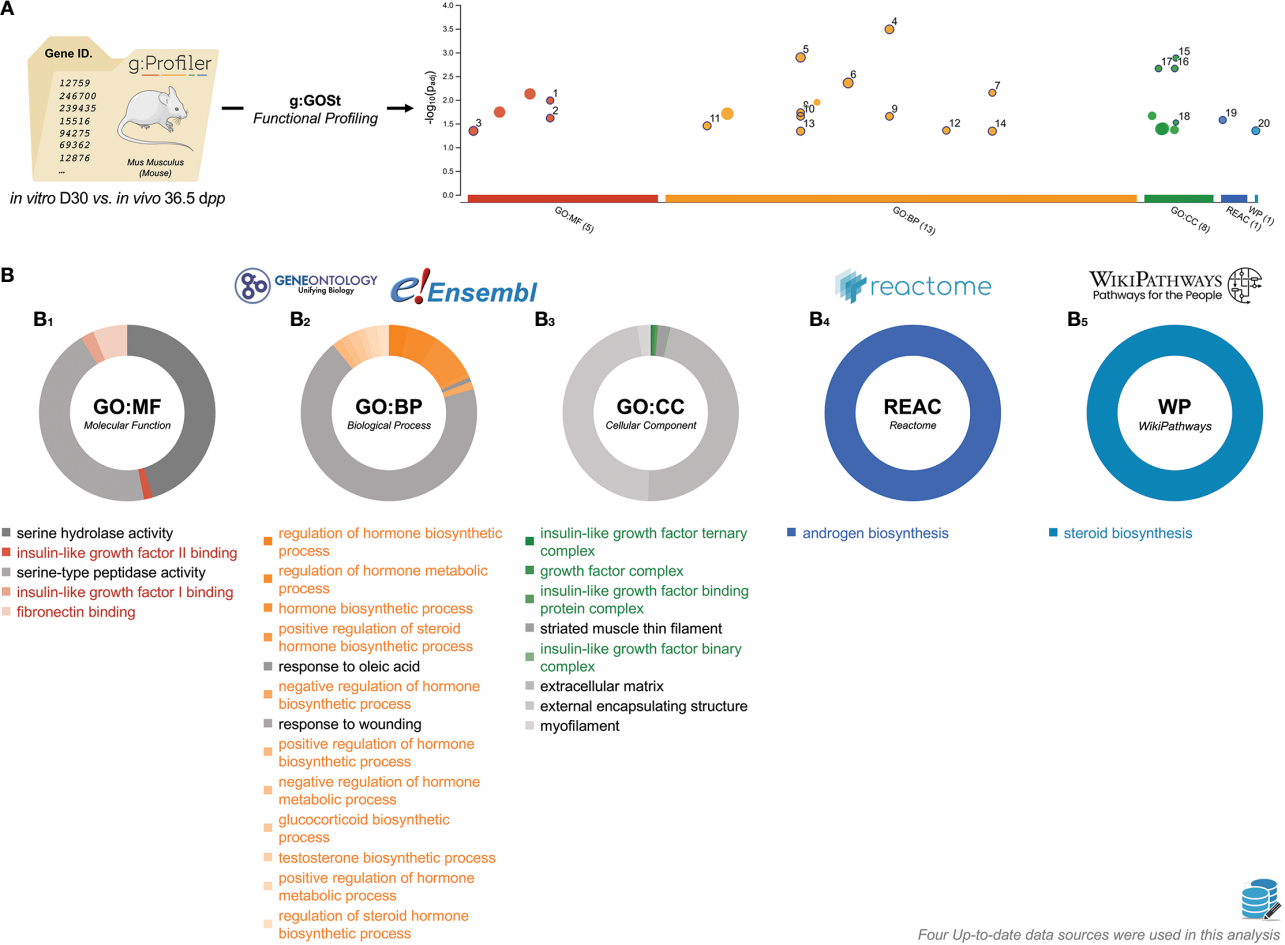
Characterization of *in vitro* D30 *vs*. *in vivo* 36.5 d*pp* gene lists. **(A)** Manhattan plot illustrating the functional enrichment analysis on *in vitro* D30 (n=4) *vs*. *in vivo* 36.5 d*pp* (n=3) input gene list. The x-axis represents functional terms that are grouped and colour-coded by data sources. The circle sizes are in accordance with the corresponding term size (*i*.*e*., larger terms have larger circles). The GO Terms locations on the x-axis is fixed and GO Terms from the same GO subtree are located closer to each other. The GO Terms lists were presented as -log_10_(p_adj_) enrichment score calculated using the formula log_10_ (FDR). **(B)** Pie chart representation of D30 *vs*. 36.5 d*pp* GO Terms **(B_1_**, molecular function; **B_2_**, biological process; **B_3_**, cellular component**)**, reactome **(B_4_)**, and WikiPathways **(B_5_)** data sources. GO Terms with the highest p_adj_ are located at noon. The surface taken by GO Terms is relative to the proportion of the number of times they were related within DEGs studied. The data can be consulted at https://biit.cs.ut.ee/gplink/l/humNT-dmTT. dpp, days *postpartum*; FDR, false discovery rate; GO, gene onthology.

Taking into account the previous results, we postulated that there would be an abnormal kinetic in the expression of genes related to late spermatogenesis at the end of the first *in vitro* spermatogenic wave. In order to understand the mechanisms that differ at the end of testicular explants in organotypic culture, an extensive g:GOSt analysis was conducted between D30 and 36.5 d*pp* (**Figure 8A**). This analysis showed that the most impacted pathways were related to two principal biological mechanisms: (i) insulin-like growth factor (IGF) binding (molecular function, **Figure 8B_1_**) and IGF complex (cellular component, **Figure 8B_3_**) and (ii) hormones and steroids (biological process, **Figure 8B_2_**), androgen biosynthesis (reactome, **Figure 8B_4_**), and steroid biosynthesis (wikipathways, **Figure 8B_5_**).

## Discussion

The purpose of the present study was to reveal unique characteristics of *in vitro* spermatogenesis throughout the first spermatogenic wave. Indeed, although interphase gas-liquid *in vitro* culture has shown to be a promising approach to generate elongated spermatids from prepubertal mice testis, complete *in vitro* spermatogenesis has not yet been accomplished in human, delaying its use in clinics for fertility restoration in survivors of childhood cancers.

Concerning the impact of cryopreservation, only 25 specific genes were identified as differentially expressed between cryopreserved and fresh tissues, indicating that freezing procedures have a minimal impact on the gene expression of testicular tissue. These data confirm what has been observed previously for the *in vitro* maturation of cryopreserved procedures in mouse (17, 18, 37) and rat (38), giving promising prospects to patients who have undergone a fertility preservation.

Although it was shown recently that testicular function in cultured postnatal mouse testis fragments is similar to that of animals during the first wave of spermatogenesis (39), numerous pathways are dysregulated in culture. At the very beginning of the culture, mechanisms of response to stress, immune response and wound healing are generated. This tissue response is in reaction to the cutting of prepubertal testicular tissue during culture. Indeed, any process that result in a change in state or activity of a cell or an organism (in terms of movement, secretion, enzyme production, gene expression, *etc*.) because of a stimulus indicating damage to the organism. A microarray analysis had already pointed out inadequate spermatogenesis and immediate radical immune reactions during a 14 days organ culture *in vitro* with the use of PDMS-ceiling chip (21). At last, oxidative stress mechanisms are manifested during culture, presumably inherent to the gas-liquid interphase system, as already shown in a previous study (40). Moreover, it has been shown that *in vitro* spermatogenesis slows down before the pachytene spermatocyte I stage in mice (21) and rats (30). This abnormal kinetic throughout the second half of the first spermatogenesis and/or blockage of spermatogenesis occurred just before and during the early meiotic phase, resulting in inefficient progression of meiosis, starting at D7 of *in vitro* culture for mice and around D16 for rat. The immune reaction, on the other hand, was drastic and overwhelming. Treatment with TAK242, an inhibitor of TLR4-NF-kB signaling pathway, ameliorated the macrophage activation which otherwise would exacerbate the inflammation in organotypic culture of mice and rat. In addition, *in vitro* system takes longer time to give rise to round spermatids as numerous cell cycle and apoptosis associated genes are differentially expressed in the spermatids when compare with *in vivo* system (41).

*Cyp17a1* expression did not increase at D4 and throughout the culture while the Leydig cell marker *Hsd3b1* is still present at levels comparable to those *in vivo*, suggesting a defective maturation of the Leydig cells *in vitro*. *Cyp17a1* encodes a member of the cytochrome P450 superfamily of enzymes that catalyze many reactions involved in drug metabolism and synthesis of cholesterol, steroids and other lipids. CYP17A1 is a key enzyme in the steroidogenic pathway that produces progestins, mineralocorticoids, glucocorticoids, androgens, and estrogens. Moreover; it has been shown in organotypic cultures of two-month-old mice testicular tissues that a decrease in steroidogenic enzyme expression, and in particular *Cyp17a1*, leads to defects in testosterone biosynthesis in Leydig cells (42). Considering that *Cyp17a1* is a Leydig cell gene that is suppressed by testosterone (43–45), there may be intratesticular accumulation of testosterone within the cultures or dysfunction in this cell type during culture. Indeed, Leydig cells are under LH control in the adult testis but they also are subject to autocrine regulation by testosterone (43, 46). The considerable overexpression of *Igfbp3* observed during culture and until the completion of the first wave of spermatogenesis could reflects an alteration in the regulation of steroidogenesis. Indeed, in human testis, the expression of *IGFBP3* is up-regulated (2.7-fold) when germ cell apoptosis is induced by intratesticular hormonal deprivation created by testosterone administration (47). IGFBP3 and BAX interaction activates germ cell apoptosis *via* the mitochondria-dependent pathway (48). IGFBP3 enters modulates nuclear hormone receptor activity by direct binding to retinoid X receptor, retinoic acid receptor (49) vitamin D receptor (50), and PPARγ (51). It seems important to point out that vitamin D3 is a secosteroid related to testosterone, cholesterol and cortisol necessary for steroidogenesis, which is essential for spermatogenesis. Besides, IGFBP-3 interacts with DNA-dependent protein kinase within the nucleus to promote the repair of DNA damage (52). The cell survival peptide humanin (which has a homologue in the rat: rattin) would be linked to the IGFBP3 protein in mice (53) and regulates the apoptosis of male germ cells when *Igfbp3* is overexpressed (47), again possibly due to an accumulation of testosterone (54).

At D16, a very significant overexpression of *Vldlr* was observed. VLDL serve to transport cholesterol that has been synthesized by the liver while chylomicrons transport cholesterol from the diet. It has been shown that chylomicrons can also bring into testicular cells retinyl ester and Rol, precursors of all-*trans*-retinoic acid (a*t*RA), which are essential for the entry into meiosis of spermatogonia *in vivo* and during the during organotypic culture *in vitro* (16, 55). It has been shown that overexpression of the *Vldlr* gene in a transgenic mouse resulted in atrophic and giant cells (56); a phenomenon that has also been observed, sometimes in large numbers, in *in vitro* testicular explants when there is a lack of a*t*RA (55). The addition of α-tocopherol during freezing could have had an impact on the regulation of VLDL, as has been demonstrated in rats (57). However, to avoid any such problem, we were rinsed tissues after freezing and cultured without the addition of vitamin E. At the end of the first spermatogenic wave, it was observed an important dysregulation of the oleic acid for testicular explants culture *in vitro*. Monounsaturated fat consumption has been associated with decreased low-density lipoprotein cholesterol, and possibly with increased high-density lipoprotein cholesterol.

Between D16 and D30, an important down-regulation of *Rps3a3* was observed. In the testicular tissue, this pseudogene is expressed in spermatocytes (6 weeks-old mouse; 58) and contradictory results on its presence in spermatids (6 weeks-old mouse; 58) or absence (8-10 weeks-old mouse; 59) were published. *Rps3a3* encodes a ribosomal protein that is a component of the 40S subunit. The top phenotype for *RPS3A* gene from catalog of human genome-wide association studies (GWAS) is “high density lipoprotein cholesterol measurement” [Mean Score=8.2]. Given that steroid hormones are made from cholesterol (60), this appears to be another strong indicator to a dysregulation of steroidogenesis during *in vitro* spermatogenesis.

In conclusion, through a combination of histological and deep transcriptomic analyses, this study confirms that testicular tissue freezing has very little impact on gene expression. The low haploid cells yield observed at the end of the first spermatogenic wave in mice is due to an abnormal kinetic throughout the second half of the first spermatogenesis and/or blockage in cultured testicular explants. These data are encouraging, especially for patients who have undergone prepubertal testicular tissue preservation in the attempt to restore their fertility. We uncovered the dysregulation of many genes and revealed that disorganized steroidogenic pathway and probably inflammation could be at the origin of the differences observed between *in vivo* and physiological controls. The transcriptomic data obtained in this study open the way to further optimization of organotypic culture procedures.

## Data availability statement

The original contributions presented in the study are publicly available. This data can be found here: https://www.ncbi.nlm.nih.gov/geo/query/acc.cgi?acc=GSE221333. All data are also conveniently accessible through the ReproGenomics Viewer (61, 62).

## Ethics statement

The animal study was reviewed and approved by Réseaux SBEA & C2EA. Written informed consent was obtained from the owners for the participation of their animals in this study.

## Author contributions

LD, conceptualization, methodology, formal analysis, software, investigation, writing – original draft preparation, writing – review and editing, visualization, supervision, project administration, funding acquisition, HL and FC, software, formal analysis, data curation, visualization, LH, LM and FB, writing – review and editing; AR-F, writing – review and editing, funding acquisition; CR, validation; NR, conceptualization, validation, writing – review and editing, supervision, funding acquisition. All authors contributed to the article and approved the submitted version.

## Footnotes

1
https://www.ncbi.nlm.nih.gov/sra

2
https://www.ensembl.org/index.html

3
http://geneontology.org

4
https://reactome.org

5
https://www.wikipathways.org/index.php/WikiPathways

6
http://www.bioconductor.org/packages/release/bioc/html/DESeq2.html

7
https://biit.cs.ut.ee/gprofiler/gost

8
https://www.R-project.org/

## References

[1] Chung SS Wolgemuth DJ. Role of retinoid signaling in the regulation of spermatogenesis. Cytogenet Genome Res (2004) 105(2-4):189–202. doi: 10.1159/000078189 15237207 PMC3803148

[2] Russell LD Ettlin RA Hikim APS Clegg ED. Histological and histopathological evaluation of the testis. Int J Androl. (1993) 16:83e83. doi: 10.1111/j.1365-2605.1993.tb01156.x

[3] Bartlett JM Kerr JB Sharpe RM. The effect of selective destruction and regeneration of rat leydig cells on the intratesticular distribution of testosterone and morphology of the seminiferous epithelium. J Androl. (1986) 7(4):240–53. doi: 10.1002/j.1939-4640.1986.tb00924.x 3745011

[4] Zirkin BR Papadopoulos V. Leydig cells: formation, function, and regulation. Biol Reprod (2018) 99(1):101–11. doi: 10.1093/biolre/ioy059 PMC6044347 29566165

[5] França LR Hess RA Dufour JM Hofmann MC Griswold MD. The sertoli cell: one hundred fifty years of beauty and plasticity. Andrology. (2016) 4(2):189–212. doi: 10.1111/andr.12165 26846984 PMC5461925

[6] Griswold MD. 50 years of spermatogenesis: Sertoli cells and their interactions with germ cells. Biol Reprod (2018) 99(1):87–100. doi: 10.1093/biolre/ioy027 29462262 PMC7328471

[7] Hanukoglu I. Steroidogenic enzymes: structure, function, and role in regulation of steroid hormone biosynthesis. J Steroid Biochem Mol Biol (1992) 43(8):779–804. doi: 10.1016/0960-0760(92)90307-5 22217824

[8] Shima JE McLean DJ McCarrey JR Griswold MD. The murine testicular transcriptome: Characterizing gene expression in the testis during the progression of spermatogenesis. Biol Reprod (2004) 71:319–30. doi: 10.1095/biolreprod.103.026880 15028632

[9] Picton HM Wyns C Anderson RA Goossens E Jahnukainen K Kliesch S. A European perspective on testicular tissue cryopreservation for fertility preservation in prepubertal and adolescent boys. Hum Reprod (2015) 30(11):2463–75. doi: 10.1093/humrep/dev190 26358785

[10] Delessard M Saulnier J Rives A Dumont L Rondanino C Rives N. Exposure to chemotherapy during childhood or adulthood and consequences on spermatogenesis and Male fertility. Int J Mol Sci (2020) 21(4):1454. doi: 10.3390/ijms21041454 32093393 PMC7073108

[11] Wyns C Curaba M Vanabelle B Van Langendonckt A Donnez J. Options for fertility preservation in prepubertal boys. Hum Reprod Update. (2010) 16(3):312–28. doi: 10.1093/humupd/dmp054 20047952

[12] Arregui L Dobrinski I. Xenografting of testicular tissue pieces: 12 years of an *in vivo* spermatogenesis system. Reproduction. (2014) 148(5):R71–84. doi: 10.1530/REP-14-0249 PMC4277018 25150043

[13] Devi L Pothana L Goel S. Dysregulation of angiogenesis-specific signalling in adult testis results in xenograft degeneration. Sci Rep (2017) 7(1):2605. doi: 10.1038/s41598-017-02604-4 28572601 PMC5454001

[14] Hou M Andersson M Eksborg S Söder O Jahnukainen K. Xenotransplantation of testicular tissue into nude mice can be used for detecting leukemic cell contamination. Hum Reprod (2007) 22(7):1899–906. doi: 10.1093/humrep/dem085 17452397

[15] Sato T Katagiri K Yokonishi T Kubota Y Inoue K Ogonuki N. *In vitro* production of fertile sperm from murine spermatogonial stem cell lines. Nat Commun (2011) 2:472. doi: 10.1038/ncomms1478 21915114

[16] Arkoun B Dumont L Milazzo JP Way A Bironneau A Wils J. Retinol improves *in vitro* differentiation of pre-pubertal mouse spermatogonial stem cells into sperm during the first wave of spermatogenesis. PLoS One (2015) 10(2):e0116660. doi: 10.1371/journal.pone.0116660 25714609 PMC4340963

[17] Dumont L Arkoun B Jumeau F Milazzo JP Bironneau A Liot D. Assessment of the optimal vitrification protocol for pre-pubertal mice testes leading to successful *in vitro* production of flagellated spermatozoa. Andrology. (2015) 3(3):611–25. doi: 10.1111/andr.12042 26013105

[18] Arkoun B Dumont L Milazzo JP Rondanino C Bironneau A Wils J. Does soaking temperature during controlled slow freezing of pre-pubertal mouse testes influence course of *in vitro* spermatogenesis? Cell Tissue Res (2016) 364(3):661–74. doi: 10.1007/s00441-015-2341-2 26714728

[19] Yokonishi T Sato T Komeya M Katagiri K Kubota Y Nakabayashi K. Offspring production with sperm grown *in vitro* from cryopreserved testis tissues. Nat Commun (2014) 5, 4320. doi: 10.1038/ncomms5320 24984101

[20] Laiho A Kotaja N Gyenesei A Sironen A. Transcriptome profiling of the murine testis during the first wave of spermatogenesis. PLoS One (2013) 8(4):e61558. doi: 10.1371/journal.pone.0061558 23613874 PMC3629203

[21] Abe T Nishimura H Sato T Suzuki H Ogawa T Suzuki T. Transcriptome analysis reveals inadequate spermatogenesis and immediate radical immune reactions during organ culture *in vitro* spermatogenesis. Biochem Biophys Res Commun (2020) 530(4):732–8. doi: 10.1016/j.bbrc.2020.06.161 32782148

[22] Abe T Nishimura H Sato T Suzuki H Ogawa T Suzuki T. Time-course microarray transcriptome data of *in vitro* cultured testes and age-matched *in vivo* testes. Data Brief. (2020) 33):106482. doi: 10.1016/j.dib.2020.106482 33241095 PMC7674299

[23] Yang QE Oatley JM. Spermatogonial stem cell functions in physiological and pathological conditions. Curr Top Dev Biol (2014) 107:235–67. doi: 10.1016/B978-0-12-416022-4.00009-3 24439809

[24] Milazzo JP Vaudreuil L Cauliez B Gruel E Massé L Mousset-Siméon N. Comparison of conditions for cryopreservation of testicular tissue from immature mice. Hum Reprod (2008) 23(1):17–28. doi: 10.1093/humrep/dem355 17989070

[25] Dobin A Davis CA Schlesinger F Drenkow J Zaleski C Jha S. STAR: ultrafast universal RNA-seq aligner. Bioinformatics. (2013) 29(1):15–21. doi: 10.1093/bioinformatics/bts635 23104886 PMC3530905

[26] Pauli A Valen E Lin MF Garber M Vastenhouw NL Levin JZ. Systematic identification of long noncoding RNAs expressed during zebrafish embryogenesis. Genome Res (2012) 22(3):577–91. doi: 10.1101/gr.133009.111 PMC3290793 22110045

[27] Trapnell C Roberts A Goff L Pertea G Kim D Kelley DR. Differential gene and transcript expression analysis of RNA-seq experiments with TopHat and cufflinks. Nat Protoc (2012) 7(3):562–78. doi: 10.1038/nprot.2012.016 PMC3334321 22383036

[28] Chalmel F Lardenois A Evrard B Rolland AD Sallou O Dumargne MC. High-resolution profiling of novel transcribed regions during rat spermatogenesis. Biol Reprod (2014) 91(1):5. doi: 10.1095/biolreprod.114.118166 24740603

[29] Zimmermann C Stévant I Borel C Conne B Pitetti JL Calvel P. Research resource: the dynamic transcriptional profile of sertoli cells during the progression of spermatogenesis. Mol Endocrinol (2015) 29(4):627–42. doi: 10.1210/me.2014-1356 PMC5414748 25710594

[30] Saulnier J Chalmel F Delessard M Moutard L Pereira T Fraissinet F. Understanding the underlying molecular mechanisms of meiotic arrest during *In vitro* spermatogenesis in rat prepubertal testicular tissue. Int J Mol Sci (2022) 23(11):5893. doi: 10.3390/ijms23115893 35682573 PMC9180380

[31] Pertea M Kim D Pertea GM Leek JT Salzberg SL. Transcript-level expression analysis of RNA-seq experiments with HISAT, StringTie and ballgown. Nat Protoc (2016) 11(9):1650–67. doi: 10.1038/nprot.2016.095 PMC5032908 27560171

[32] Chalmel F Primig M. The annotation, mapping, expression and network (AMEN) suite of tools for molecular systems biology. BMC Bioinf (2008) 9:86. doi: 10.1186/1471-2105-9-86 PMC2375118 18254954

[33] Wettenhall JM Smyth GK. limmaGUI: a graphical user interface for linear modeling of microarray data. Bioinformatics. (2004) 20(18):3705–6. doi: 10.1093/bioinformatics/bth449 15297296

[34] Lê S Josse J Husson F. FactoMineR: An r package for multivariate analysis. J Stat Software (2008) 25(1):1–18. doi: 10.18637/jss.v025.i01

[35] Anders S Pyl PT Huber W. HTSeq–a Python framework to work with high-throughput sequencing data. Bioinformatics (2015) 31(2):166–9. doi: 10.1093/bioinformatics/btu638 PMC4287950 25260700

[36] Benjamini Y Hochberg Y. Controlling the false discovery rate: a practical and powerful approach to multiple testing. J R Stat Soc Ser B (1995) 57(1):289–300. doi: 10.1111/j.2517-6161.1995.tb02031.x

[37] Dumont L Chalmel F Oblette A Berby B Rives A Duchesne V. Evaluation of apoptotic- and autophagic-related protein expressions before and after IVM of fresh, slow-frozen and vitrified pre-pubertal mouse testicular tissue. Mol Hum Reprod (2017) 23(11):738–54. doi: 10.1093/molehr/gax054 29040674

[38] Saulnier J Oblette A Delessard M Dumont L Rives A Rives N. Improving freezing protocols and organotypic culture: A histological study on rat prepubertal testicular tissue. Ann BioMed Eng. (2021) 49(1):203–18. doi: 10.1007/s10439-020-02535-8 32440757

[39] Pence LM Schmitt TC Beger RD Del Valle PL Nakamura N. Testicular function in cultured postnatal mouse testis fragments is similar to that of animals during the first wave of spermatogenesis. Birth Defects Res (2019) 111(5):270–80. doi: 10.1002/bdr2.1451 30703285

[40] Arkoun B Galas L Dumont L Rives A Saulnier J Delessard M. Vitamin e but not GSH decreases reactive oxygen species accumulation and enhances sperm production during *In vitro* maturation of frozen-thawed prepubertal mouse testicular tissue. Int J Mol Sci (2019) 20(21):5380. doi: 10.3390/ijms20215380 31671759 PMC6861907

[41] Yao J Zuo H Gao J Wang M Wang D Li X. The effects of IGF-1 on mouse spermatogenesis using an organ culture method. Biochem Biophys Res Commun (2017) 491(3):840–7. doi: 10.1016/j.bbrc.2017.05.125 28552527

[42] Chen M Wang X Wang Y Zhang L Xu B Lv L. Wt1 is involved in leydig cell steroid hormone biosynthesis by regulating paracrine factor expression in mice. Biol Reprod (2014) 90(4):71. doi: 10.1095/biolreprod.113.114702 24571983

[43] Burgos-Trinidad M Youngblood GL Maroto MR Scheller A Robins DM Payne AH. Repression of cAMP-induced expression of the mouse P450 17 alpha-hydroxylase/C17-20 lyase gene (Cyp17) by androgens. Mol Endocrinol (1997) 11(1):87–96. doi: 10.1210/mend.11.1.9871 8994191

[44] Ohsako S Kubota K Kurosawa S Takeda K Qing W Ishimura R. Alterations of gene expression in adult male rat testis and pituitary shortly after subacute administration of the antiandrogen flutamide. J Reprod Dev (2003) 49(4):275–90. doi: 10.1262/jrd.49.275 14967920

[45] Zhou Q Shima JE Nie R Friel PJ Griswold MD. Androgen-regulated transcripts in the neonatal mouse testis as determined through microarray analysis. Biol Reprod (2005) 72(4):1010–9. doi: 10.1095/biolreprod.104.035915 15601916

[46] O’Shaughnessy PJ Johnston H Willerton L Baker PJ. Failure of normal adult leydig cell development in androgen-receptor-deficient mice. J Cell Sci (2002) 115(Pt 17):3491–6. doi: 10.1242/jcs.115.17.3491 12154079

[47] Lue Y Swerdloff R Liu Q Mehta H Hikim AS Lee KW. Opposing roles of insulin-like growth factor binding protein 3 and humanin in the regulation of testicular germ cell apoptosis. Endocrinology. (2010) 151(1):350–7. doi: 10.1210/en.2009-0577 19952275

[48] Jia Y Lee KW Swerdloff R Hwang D Cobb LJ Sinha Hikim A. Interaction of insulin-like growth factor-binding protein-3 and BAX in mitochondria promotes male germ cell apoptosis. J Biol Chem (2010) 285(3):1726–32. doi: 10.1074/jbc.M109.046847 PMC2804330 19887447

[49] Liu B Lee HY Weinzimer SA Powell DR Clifford JL Kurie JM. Direct functional interactions between insulin-like growth factor-binding protein-3 and retinoid X receptor-alpha regulate transcriptional signaling and apoptosis. J Biol Chem (2000) 275(43):33607–13. doi: 10.1074/jbc.M002547200 10874028

[50] Ikezoe T Tanosaki S Krug U Liu B Cohen P Taguchi H. Insulin-like growth factor binding protein-3 antagonizes the effects of retinoids in myeloid leukemia cells. Blood. (2004) 104(1):237–42. doi: 10.1182/blood-2003-07-2203 15026318

[51] Chan SS Schedlich LJ Twigg SM Baxter RC. Inhibition of adipocyte differentiation by insulin-like growth factor-binding protein-3. Am J Physiol Endocrinol Metab (2009) 296(4):E654–663. doi: 10.1152/ajpendo.90846.2008 19141684

[52] Lin MZ Marzec KA Martin JL Baxter RC. The role of insulin-like growth factor binding protein-3 in the breast cancer cell response to DNA-damaging agents. Oncogene. (2014) 33(1):85–96. doi: 10.1038/onc.2012.538 23178489

[53] Ikonen M Liu B Hashimoto Y Ma L Lee KW Niikura T. Interaction between the Alzheimer's survival peptide humanin and insulin-like growth factor-binding protein 3 regulates cell survival and apoptosis. Proc Natl Acad Sci USA (2003) 100 (22):13042–7. doi: 10.1073/pnas.2135111100 PMC240741 14561895

[54] Lue Y Wang C Cui Y Wang X Sha J Zhou Z. Levonorgestrel enhances spermatogenesis suppression by testosterone with greater alteration in testicular gene expression in men. Biol Reprod (2009) 80(3):484–92. doi: 10.1095/biolreprod.108.070839 PMC6354713 19074003

[55] Dumont L Oblette A Rondanino C Jumeau F Bironneau A Liot D. Vitamin a prevents round spermatid nuclear damage and promotes the production of motile sperm during *in vitro* maturation of vitrified pre-pubertal mouse testicular tissue. Mol Hum Reprod (2016) 22(12):819–32. doi: 10.1093/molehr/gaw063 27671755

[56] Tacken PJ van der Zee A Beumer TL Florijn RJ Gijpels MJ Havekes LM. Effective generation of very low density lipoprotein receptor transgenic mice by overlapping genomic DNA fragments: high testis expression and disturbed spermatogenesis. Transgenic Res (2001) 10(3):211–21. doi: 10.1023/A:1016682520887 11437278

[57] Kato N Momota Y Kusuhara T. Changes in distribution of alpha-tocopherol and cholesterol in serum lipoproteins and tissues of rats by dietary PCB and dietary level of protein. J Nutr Sci Vitaminol (Tokyo). (1989) 35(6):655–60. doi: 10.3177/jnsv.35.655 2517511

[58] Bao J Vitting-Seerup K Waage J Tang C Ge Y Porse BT. UPF2-dependent nonsense-mediated mRNA decay pathway is essential for spermatogenesis by selectively eliminating longer 3'UTR transcripts. PLoS Genet (2016) 12(5):e1005863. doi: 10.1371/journal.pgen.1005863 27149259 PMC4858225

[59] Anuar ND Kurscheid S Field M Zhang L Rebar E Gregory P. Gene editing of the multi-copy H2A.B gene and its importance for fertility. Genome Biol (2019) 20(1):23. doi: 10.1186/s13059-019-1633-3 30704500 PMC6357441

[60] Miller WL Bose HS. Early steps in steroidogenesis: intracellular cholesterol trafficking. J Lipid Res (2011) 52(12):2111–35. doi: 10.1194/jlr.R016675 PMC3283258 21976778

[61] Darde TA Sallou O Becker E Evrard B Monjeaud C Le Bras Y. The ReproGenomics viewer: an integrative cross-species toolbox for the reproductive science community. Nucleic Acids Res (2015) 43(W1):W109–116. doi: 10.1093/nar/gkv345 PMC4489245 25883147

[62] Darde TA Lecluze E Lardenois A Stévant I Alary N Tüttelmann F. The ReproGenomics viewer: a multi-omics and cross-species resource compatible with single-cell studies for the reproductive science community. Bioinformatics. (2019) 35(17):3133–9. doi: 10.1093/bioinformatics/btz047 30668675

