# Supplementary material for: Throughout in vitro first spermatogenic wave: Next-generation sequencing gene expression patterns of fresh and cryopreserved prepubertal mice testicular tissue explants
Source: Front Endocrinol (Lausanne). 2023 Mar 17;14:1112834. doi: 10.3389/fendo.2023.1112834 (PMC10063980; doi:10.3389/fendo.2023.1112834)
Supplement: Supplementary file 1 [file DataSheet_1.pdf]

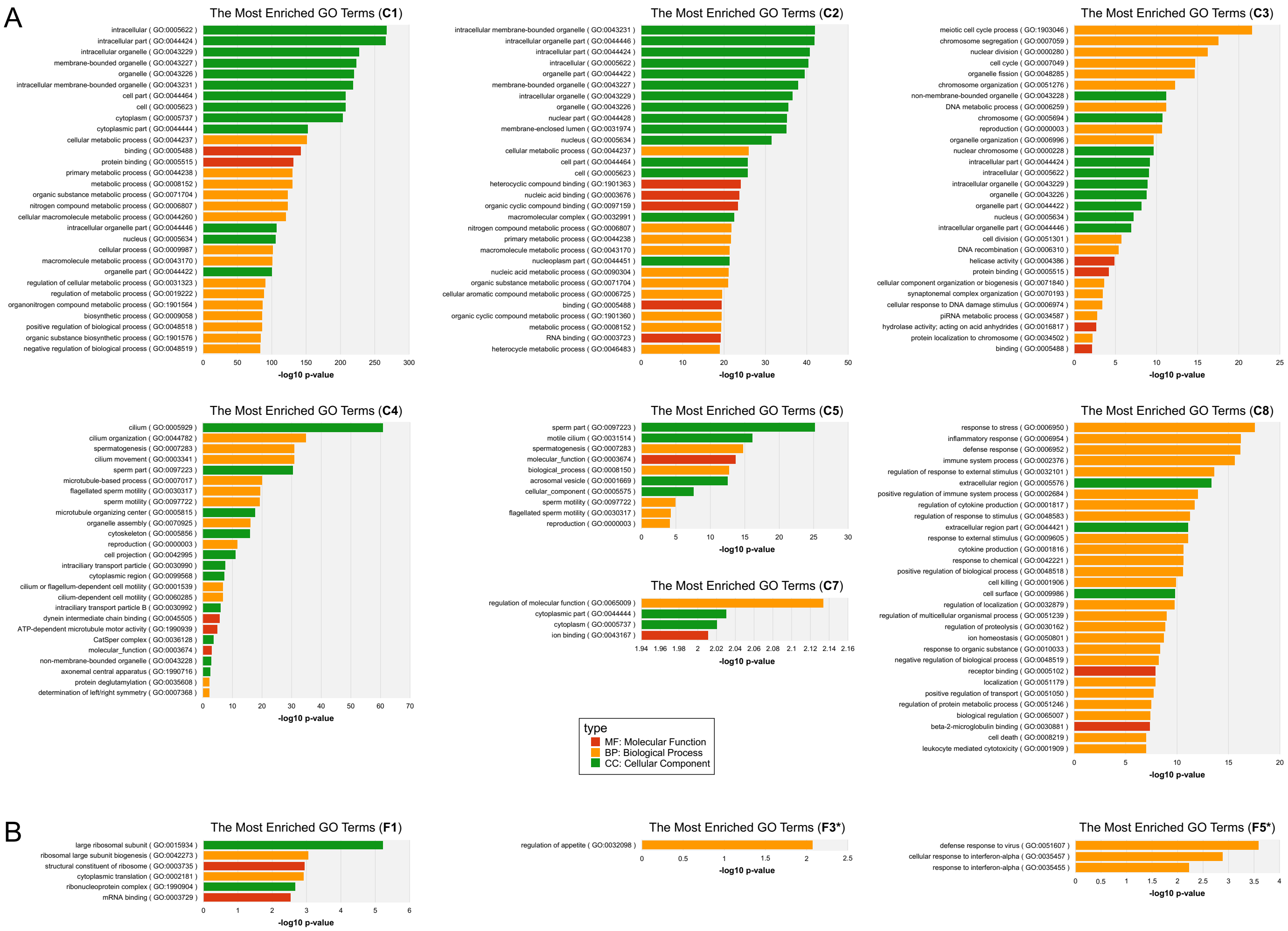

Supplementary Figure S1. Functional GO Terms enrichments of DEGs categorized by their gene expression dynamics of each category. Bar charts of the distribution of corresponding GO Terms of biological processes, molecular functions, and cellular components in which transcripts were involved across the entire sample set for the evaluation of *in vitro* culture (**A**) and freezing procedure (**B**). The 30 most representative GO Terms were presented by pattern.

DEGs, differentially expressed genes; GO, gene ontology.

frontiers
