# Supplementary material for: Throughout in vitro first spermatogenic wave: Next-generation sequencing gene expression patterns of fresh and cryopreserved prepubertal mice testicular tissue explants
Source: Front Endocrinol (Lausanne). 2023 Mar 17;14:1112834. doi: 10.3389/fendo.2023.1112834 (PMC10063980; doi:10.3389/fendo.2023.1112834)
Supplement: Supplementary file 2 [file DataSheet_2.pdf]

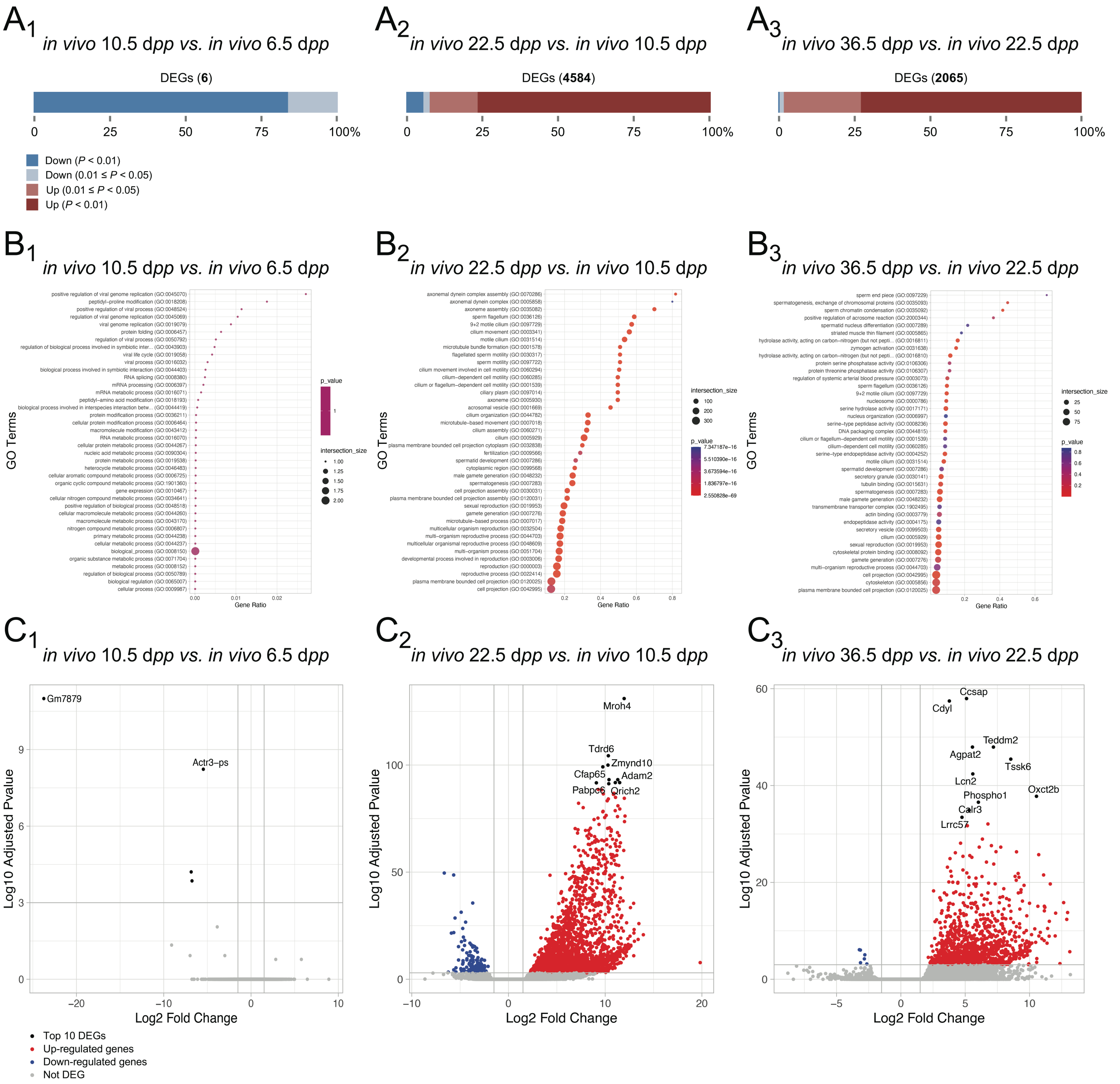

D1

Gene	log_2-fold change	p_{adj}
Gm7879	-23.73839467	1.00E-11
Actr3-ps	-5.464712497	5.90E-09
Amd-ps4	-6.855798634	6.22E-05
Gm8113	-6.763196298	1.40E-04

D2

Gene	log_2-fold change	p_{adj}
Mroh4	11.96155825	9.51E-132
Tdrd6	10.32721494	4.72E-105
Zmynd10	10.28848936	1.09E-100
Cfap65	9.756557473	7.35E-100
Ccdc60	10.39550588	6.82E-94
*1700123L14Rik**	11.30215212	6.82E-94
Adam2	11.49694599	1.60E-92
Qrich2	11.04554420	1.60E-92
Pabpc6	9.087558392	2.06E-92
Adam32	10.37133885	5.38E-92

D3

Gene	log_2-fold change	p_{adj}
Ccsap	5.103262153	1.09E-58
Cdyl	3.766832470	3.51E-58
Agpat2	5.562369686	1.14E-48
Teddm2	7.191648895	1.14E-48
Tssk6	8.540596298	3.53E-46
Lcn2	5.589264880	3.98E-43
Oxct2b	10.53748955	1.66E-38
Phospho1	6.024095008	2.79E-37
Calr3	5.307788284	1.26E-35
Lrrc57	4.748962518	3.61E-34

Supplementary Figure S2. Gene-level differential expression analysis throughout the *in vivo* kinetics of the first spermatogenic wave. (**A**) The proportion of significantly DEGs are shown for the kinetic timepoints of 10.5 d*pp* *vs.* 6.5 d*pp* (**A_1**), 22.5 d*pp* *vs.* 10.5 d*pp* (**A_2**), and 36.5 d*pp* *vs*. 22.5 d*pp* (**A_3**). The number of DEGs for each comparison is available in order to have an appreciation of the difference in gene expression between the two conditions compared. (**B_{1-3}**) Corresponding GO enrichment dot plot. The 40 GO processes with the largest gene ratios are plotted in order of gene ratio. The size of the dots represent the number of genes in the significant DEGs associated with the GO Terms and the color of the dots represent the p_{adj} values. (**C_{1-3}**) Volcano plots compare the amount of gene expression change to the significance of that change (here plotted as the

frontiers

log_{10} transformation of the multiple test p_{adj} value), with each point representing a single gene. The top 10 gene candidates are highlighted in black and by text labeling. The two marginal plots showing the distributions of the log_2-fold changes and negative log10 p_{adj} values are used to show cutoff choices and trade-offs. (**D_{1-3}**) Top ten DEGs with corresponding log_2-fold change and p_{adj} value.

*BioType Conflict, biotypes are flagged as conflicting when annotations from multiple sources for the same genome feature in the same strain are different (biotype annotations that differ among different strains for the equivalent genome feature are considered polymorphisms, not conflicts); DEGs, differentially expressed genes.

Main implications of principal top 10 DEGs

• ***In vivo* 22.5 d*pp vs. in vivo* 10.5 d*pp*.** Despite its unknown functions, *Mroh4* shows testis-specific expression in EST Profile (UniGene) and Gene Expression Atlas (EMBLEBI). Within testicular cell types, *Mroh4* is enriched in spermatocytes and spermatids in mice (Zhou *et al*., 2017). In mice, *Tdrd6* is known to be gametogenesis stage- and male-specific expressed, essential for spermiogenesis, directly in interaction with the chromatoid bodies structure components Mili and Miwi (Vasileva *et al*., 2009), and mediates early steps of spliceosome maturation in primary spermatocytes, more specifically in prophase I (Akpınar *et al*., 2017). *Zmynd10*, mainly expressed in testicular tissue, is a gene essential for proper axonemal assembly of inner and outer dynein arms in humans and flies (Moore *et al*., 2013). In human, *CFAP65* is essential for acrosome formation and flagellum assembly (Wang *et al*., 2019) and is required in the acrosome biogenesis and mitochondrial sheath assembly during spermiogenesis (Wang *et al*., 2021). The role of testis-specific *1700123L14Rik* gene (*Nup50B*) in spermatogenesis is currently unknown but its presence has already been reported by several studies within the mice testicular tissue and could be related to pachytene spermatocytes I (Wang *et al*., 2016). It has been previously reported that a *Nup50* knockout mouse is not viable but that fibroblasts derived from mouse embryos can be kept in culture (Smitherman *et al*., 2000). Although referred to as a pseudogene, *1700123L14Rik* is effectively expressed as indicated in the expression atlas (Papatheodorou *et al*., 2020) and the mouse genome database (Bult *et al*., 2019) and has been previously named *Nup50rel* (Smitherman *et al*., 2000). *Nup50B* might substitute the canonical *Nup50* paralog at least in mouse fibroblasts. Together, these data indicate that *Nup50* has a crucial function in nuclear pore complexes assembly during mitotic exit (Holzer *et al*., 2021). *Adam2* and *Adam32* are genes predominantly expressed in the testis coding to sperm surface membrane proteins that are involved in sperm-egg plasma membrane adhesion and fusion during fertilization (Nishimura *et al*, 2007; Choi *et al*., 2003). In human, *QRICH2* has an essential role in the formation of sperm flagella and flagellar structure maintenance. Mainly expressed in testicular tissue, the QRICH2 protein has been shown to acts as a suppressor of ubiquitination and degradation of proteins involved in flagellar development and motility (Shen *et al*., 2019). Despite its unknown specific function, *Pabpc6* is a highly conserved gene across evolution in vertebrates and invertebrates and seems to be involved in germ cell development (Fouchécourt *et al*., 2019).

• ***In vivo* 36.5 d*pp vs. in vivo* 22.5 d*pp*.** *Ccsap* is a gene coding for a protein promoting microtubule stabilization and regulate bipolar spindle formation in mitosis, ensuring proper bipolar spindle formation and maintenance (Ohta *et al*., 2015). *Cdyl* is expressed exclusively in the testis and is implicated in infertility. Protein encoded by this gene possess a chromodomain, a motif implicated in chromatin binding and gene suppression, and a catalytic domain believed to be involved in histone acetylation (Liu *et al*., 2017). Indeed, CDYL promotes H3K27me3 methylation at DNA double strand breaks, thereby facilitating transcriptional repression at sites of DNA damage and homology-directed repair (Abu-Zhayia *et al*., 2018). *Tssk6* is a gene coding for a serine/threonine protein kinase that is required for postmeiotic chromatin remodeling and male fertility (Spiridonov *et al*., 2005). *Lcn2* encodes a protein that belongs to the lipocalin family that transport small hydrophobic molecules such as lipids, steroid hormones and retinoids (Kang *et al*., 2017; De La Chesnaye *et al*., 2020). *Oxct2b* is a haploid-specific gene regulated by a CRE-like element and bound to a testis-specific CREM isoform (Somboonthum *et al*., 2005).

Specific References for Fig. S2

Abu-Zhayia, E.R., Awwad, S.W., Ben-Oz, B.M., Khoury-Haddad, H. and Ayoub, N. (2018). CDYL1

frontiers

fosters double-strand break-induced transcription silencing and promotes homology-directed repair. J Mol Cell Biol. 10(4), 341-357.

Akpınar, M., Lesche, M., Fanourgakis, G., Fu, J., Anastassiadis, K., Dahl, A. and Jessberger, R. (2017). TDRD6 mediates early steps of spliceosome maturation in primary spermatocytes. PLoS Genet. 13(3), e1006660.

Bult, C.J., Blake, J.A., Smith, C.L., Kadin, J.A., Richardson, J.E. and Mouse Genome Database Group. (2019). Mouse Genome Database (MGD) 2019. Nucleic Acids Res. 47(D1), D801-D806.

Choi, I., Woo, J.M., Hong, S., Jung, Y.K., Kim, D.H. and Cho, C. (2003). Identification and characterization of ADAM32 with testis-predominant gene expression. Gene. 304, 151-162.

De La Chesnaye, E., Manuel-Apolinar, L., Damasio, L., Castrejón, E., López-Ballesteros, R., Revilla-Monsalve, M.C. and, Méndez, J.P. (2020). The gonadal expression pattern of lipocalin-2 and 24p3 receptor is modified in the gonads of the offspring of obese rats. Mol Med Rep. 22(2), 1409-1419.

Fouchécourt, S., Picolo, F., Elis, S., Lécureuil, C., Thélie, A., Govoroun, M., Brégeon, M., Papillier, P., Lareyre, J.J. and Monget, P. (2019). An evolutionary approach to recover genes predominantly expressed in the testes of the zebrafish, chicken and mouse. BMC Evol Biol. 19(1), 137.

Holzer, G., De Magistris, P., Gramminger, C., Sachdev, R., Magalska, A., Schooley, A., Scheufen, A., Lennartz, B., Tatarek-Nossol, M., Lue, H. et al. (2021). The nucleoporin Nup50 activates the Ran guanine nucleotide exchange factor RCC1 to promote NPC assembly at the end of mitosis. EMBO J. 40(23), e108788.

Kang, Z., Qiao, N., Tan, Z., Tang, Z. and Li, Y. Expression patterns and changes of the LCN2 gene in the testes of induced cryptorchidism and busulfan-treated mice. Syst Biol Reprod Med. 2017 Dec;63(6):364-369.

Liu, S., Yu, H., Liu, Y., Liu, X., Zhang, Y., Bu, C., Yuan, S., Chen, Z., Xie, G., Li, W. et al. (2017). Chromodomain Protein CDYL Acts as a Crotonyl-CoA Hydratase to Regulate Histone Crotonylation and Spermatogenesis. Mol Cell. 67(5), 853-866.e5.

Moore, D.J., Onoufriadis, A., Shoemark, A., Simpson, M.A., zur Lage, P.I., de Castro, S.C., Bartoloni, L., Gallone, G., Petridi, S., Woollard, W.J. et al. (2013). Mutations in ZMYND10, a gene essential for proper axonemal assembly of inner and outer dynein arms in humans and flies, cause primary ciliary dyskinesia. Am J Hum Genet. 93(2), 346-356.

Nishimura, H., Myles, D.G. and Primakoff, P. (2007). Identification of an ADAM2-ADAM3 complex on the surface of mouse testicular germ cells and cauda epididymal sperm. J Biol Chem. 282(24), 17900-17907.

Ohta, S., Hamada, M., Sato, N. and Toramoto, I. (2015). Polyglutamylated Tubulin Binding Protein C1orf96/CSAP Is Involved in Microtubule Stabilization in Mitotic Spindles. PLoS One. 10(11), e0142798.

Papatheodorou, I., Moreno, P., Manning, J., Fuentes, A.M., George, N., Fexova, S., Fonseca, N.A., Füllgrabe, A., Green, M., Huang, N. et al. (2020). Expression Atlas update: from tissues to single cells. Nucleic Acids Res. 48(D1), D77-D83.

Shen, Y., Zhang, F., Li, F., Jiang, X., Yang, Y., Li, X., Li, W., Wang, X., Cheng, J., Liu, M. et al. (2019). Loss-of-function mutations in QRICH2 cause male infertility with multiple morphological abnormalities of the sperm flagella. Nat Commun. 10(1), 433.

Smitherman, M., Lee, K., Swanger, J., Kapur, R. and Clurman, B.E. (2000). Characterization and targeted disruption of murine Nup50, a p27(Kip1)-interacting component of the nuclear pore complex. Mol Cell Biol. 20(15), 5631-5642.

Somboonthum, P., Ohta, H., Yamada, S., Onishi, M., Ike, A., Nishimune, Y. and Nozaki, M. (2005). cAMP-responsive element in TATA-less core promoter is essential for haploid-specific gene expression

in mouse testis. Nucleic Acids Res. 33(10), 3401-3411.

Spiridonov, N.A., Wong, L., Zerfas, P.M., Starost, M.F., Pack, S.D., Paweletz, C.P. and Johnson, G.R. (2005). Identification and characterization of SSTK, a serine/threonine protein kinase essential for male fertility. Mol Cell Biol. 25(10), 4250-4261.

Vasileva, A., Tiedau, D., Firooznia, A., Müller-Reichert, T. and Jessberger, R. (2008). Tdrd6 is required for spermiogenesis, chromatoid body architecture, and regulation of miRNA expression. Curr Biol. 19(8), 630-639.

Wang, L., Guo, Y., Liu, W., Zhao, W., Song, G., Zhou, T., Huang, H., Guo, X. and Sun, F. (2016). Proteomic Analysis of Pachytene Spermatocytes of Sterile Hybrid Male Mice. Biol Reprod. 95(3), 52.

Wang, W., Tu, C., Nie, H., Meng, L., Li, Y., Yuan, S., Zhang, Q., Du, J., Wang, J., Gong, F. et al. (2019). Biallelic mutations in CFAP65 lead to severe asthenoteratospermia due to acrosome hypoplasia and flagellum malformations. J Med Genet. 56(11), 750-757.

Wang, W., Tian, S., Nie, H., Tu, C., Liu, C., Li, Y., Li, D., Yang, X., Meng, L., Hu, T. et al. (2021). CFAP65 is required in the acrosome biogenesis and mitochondrial sheath assembly during spermiogenesis. Hum Mol Genet. 30(23), 2240-2254.

Zhou, L., Canagarajah, B., Zhao, Y., Baibakov, B., Tokuhiro, K., Maric, D. and Dean, J. (2017). BTBD18 Regulates a Subset of piRNA-Generating Loci through Transcription Elongation in Mice. Dev Cell. 40(5), 453-466.e5.

frontiers
