# Supplementary material for: Throughout in vitro first spermatogenic wave: Next-generation sequencing gene expression patterns of fresh and cryopreserved prepubertal mice testicular tissue explants
Source: Front Endocrinol (Lausanne). 2023 Mar 17;14:1112834. doi: 10.3389/fendo.2023.1112834 (PMC10063980; doi:10.3389/fendo.2023.1112834)
Supplement: Supplementary file 3 [file DataSheet_3.pdf]

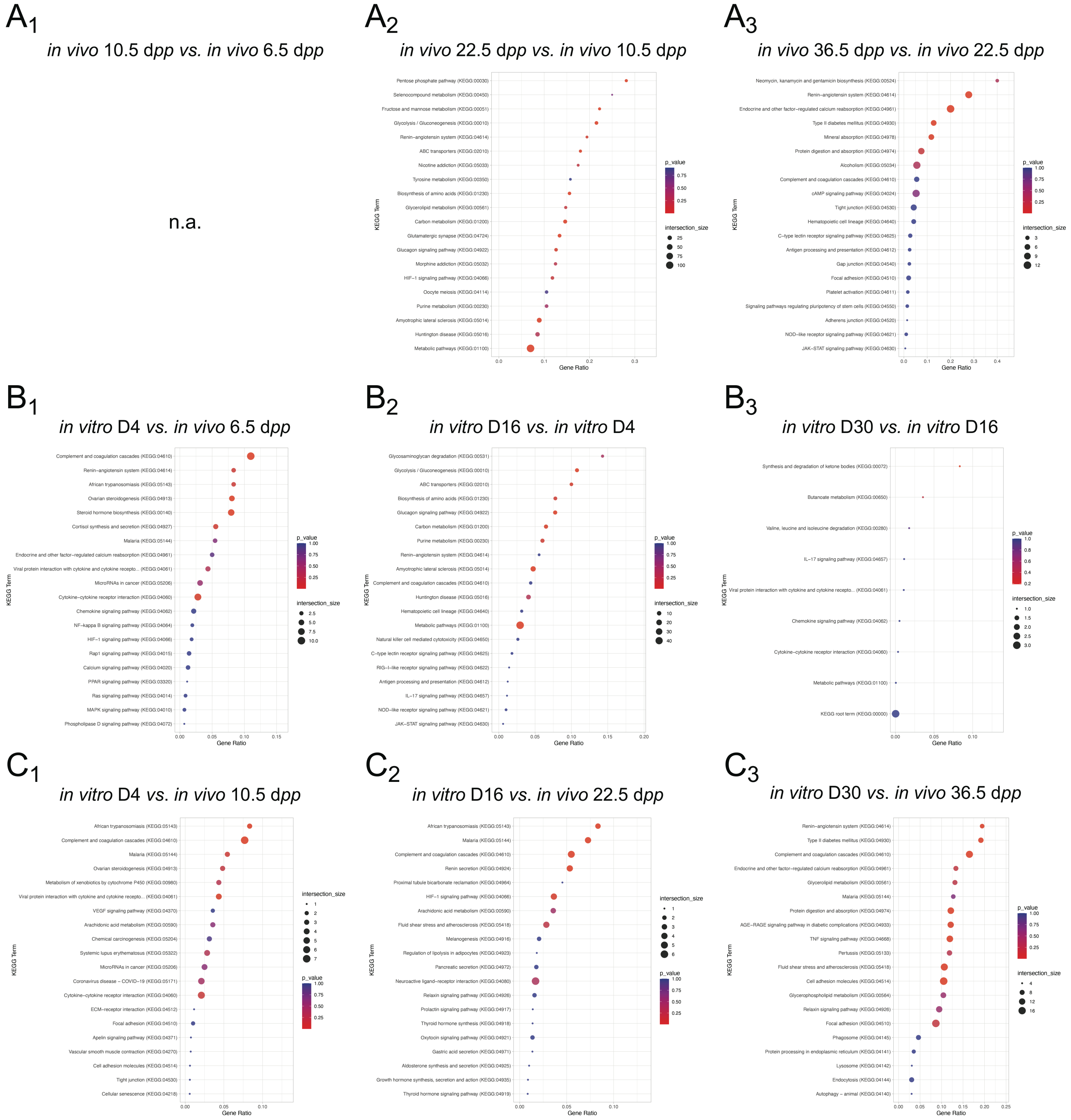

Supplementary Figure S3. KEGG pathway enrichment analysis. This analysis was used to study the significantly altered pathways for DEGs between groups. Dot plot shows the dysregulated KEGG pathways enriched for different contrasts. The size of the dot is based on gene count enriched in the pathway, and the color of the dot shows the pathway enrichment significance. The 20 GO processes with the largest gene ratios are plotted in order of gene ratio. (**A$_{1\text{-}3}$**) KEGG pathway enrichment dot plot throughout the *in vivo* kinetics of the first spermatogenic wave. For the condition 10.5 d*pp vs.* 6.5 d*pp*, no KEGG pathway could be identified because of the limited number of DEGs. (**B$_{1\text{-}3}$**) KEGG pathway enrichment dot plot throughout the *in vitro* kinetics of the first spermatogenic wave. (**C$_{1\text{-}3}$**) KEGG pathway enrichment dot plot between *in vitro* and *in vivo* age-matched testicular samples.

DEGs, differentially expressed genes, KEGG, Kyoto encyclopedia of genes and genomes, n.a., data not available.
