# Supplementary material for: Throughout in vitro first spermatogenic wave: Next-generation sequencing gene expression patterns of fresh and cryopreserved prepubertal mice testicular tissue explants
Source: Front Endocrinol (Lausanne). 2023 Mar 17;14:1112834. doi: 10.3389/fendo.2023.1112834 (PMC10063980; doi:10.3389/fendo.2023.1112834)
Supplement: Supplementary file 4 [file DataSheet_4.pdf]

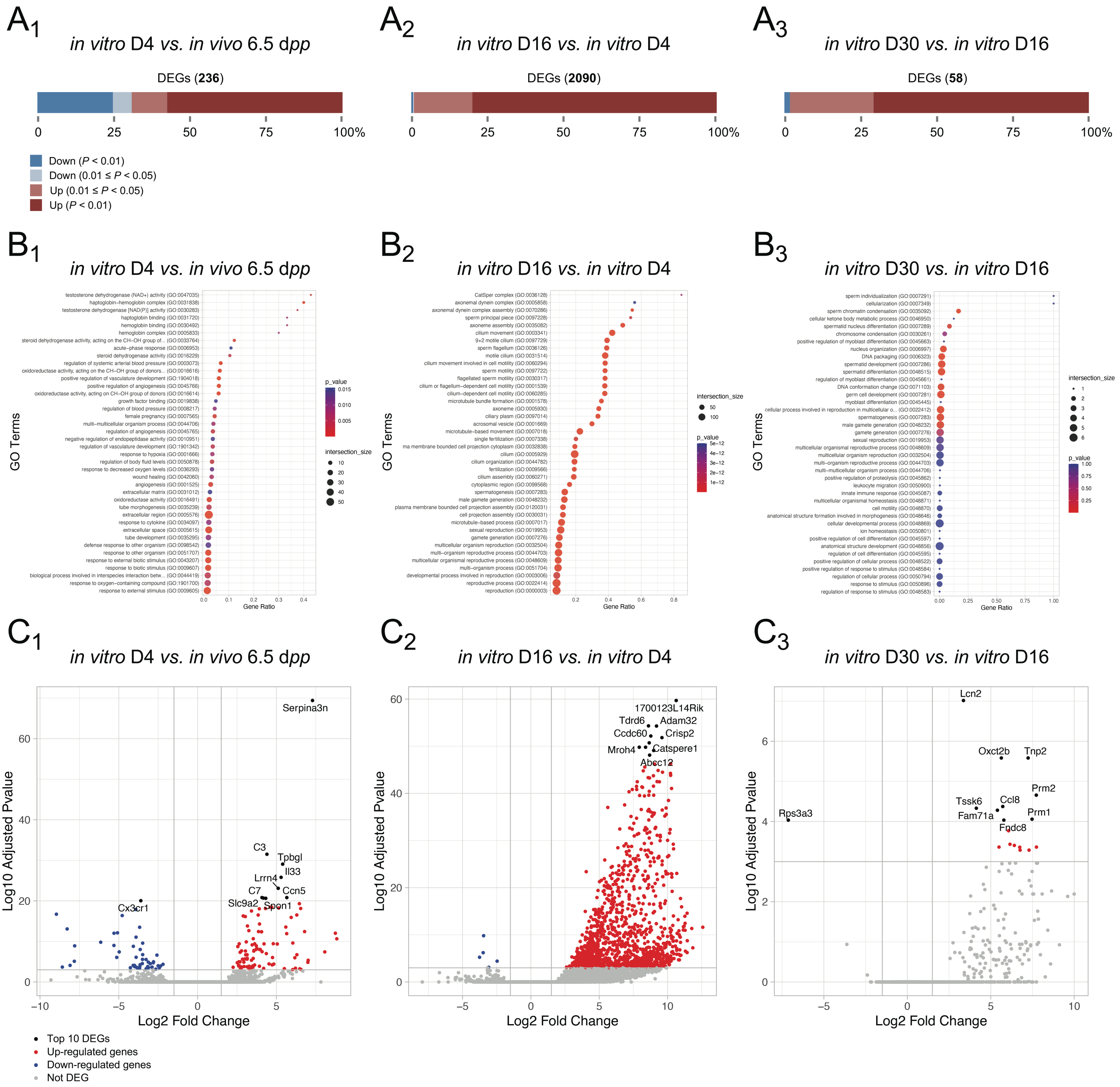

D1

Gene	log2-fold change	p_{adj}
Serpina3n	7.290392442	3.81E-70
C3	4.398405948	3.14E-32
Tpbgl	5.398050673	8.69E-30
Il33	5.297191589	1.55E-26
Lrrn4	5.115747952	7.98E-24
Ccn5	5.659965946	1.54E-21
C7	4.082993797	1.54E-21
Slc9a2	4.173599256	2.05E-21
Spon1	4.324510915	2.32E-21
Cx3cr1	-3.603404932	9.17E-21

D2

Gene	log2-fold change	p_{adj}
*1700123L14Rik**	10.63299052	2.01E-60
Tdrd6	8.603602312	4.86E-55
Adam32	9.191426689	5.28E-55
Ccdc60	8.773493307	6.86E-53
Crisp2	9.588885136	1.52E-52
Catspere1	8.657762597	1.94E-51
Mroh4	7.929953276	1.64E-50
Gm21269	8.390690487	1.64E-50
4930444F02Rik	8.985747662	7.91E-50
Abcc12	8.688149945	7.76E-49

D3

Gene	log2-fold change	p_{adj}
Lcn2	3.362252085	9.64E-08
Tnp2	7.236796178	2.61E-06
Oxct2b	5.631676490	2.61E-06
Prm2	7.726642269	2.20E-05
Ccl8	5.716861850	4.23E-05
Tssk6	4.133715345	4.67E-05
Fam71a	5.396506079	5.25E-05
Prm1	7.471844296	8.75E-05
Fndc8	5.782249903	9.24E-05
Rps3a3	-7.131679005	9.24E-05

Supplementary Figure S4. Gene-level differential expression analysis throughout the *in vitro* kinetics of the first spermatogenic wave. (**A**) The proportion of significantly DEGs are shown for the kinetic timepoints of D4 *vs*. 6.5 d*pp* (corresponding to D0) (**A$_1$**), D16 *vs*. D4 (**A$_2$**), and D30 *vs*. D16 (**A$_3$**). The number of DEGs for each comparison is available in order to have an appreciation of the difference in gene expression between the two conditions compared. (**B$_{1-3}$**) Corresponding GO enrichment dot plot. The 40 GO processes with the largest gene ratios are plotted in order of gene ratio. The size of the dots represents the number of genes in the significant DEGs associated with the GO Terms and the color of the dots represent the p_{adj} values. (**C$_{1-3}$**) Volcano plots compare the amount of gene expression change to the significance of that change (here plotted as the $\log_{10}$ transformation of the multiple test p_{adj} value),

frontiers

with each point representing a single gene. The top 10 gene candidates are highlighted in black and by text labeling. The two marginal plots showing the distributions of the log_2-fold changes and negative log10 p_{adj} values are used to show cutoff choices and trade-offs. (**D_{1-3}**) Top ten DEGs with corresponding log_2-fold change and p_{adj} value.

*BioType Conflict, biotypes are flagged as conflicting when annotations from multiple sources for the same genome feature in the same strain are different (biotype annotations that differ among different strains for the equivalent genome feature are considered polymorphisms, not conflicts); DEGs, differentially expressed genes.

Main implications of principal top 10 DEGs

• ***In vitro* D4 *vs. in vivo* 6.5 d*pp*. *Serpina3n*** is a gene coding for a protease inhibitor cellular response to cytokine stimulus and well known to reduce apoptosis and inflammation. In addition, it was shown that Sertoli cells secrete Serpina3n that inhibits granzyme B-mediated apoptosis (Sipione *et al*., 2006). *Complement component 3* (*C3*) and *C7* encodes proteins that play central roles in the activation of complement system of the innate immune system (at middle and late steps of the complement cascade, respectively) (Janeway *et al*., 2001). Their activation are required for both classical and alternative complement activation pathways. *Il33* is a member of the cytokine family that is upregulated in non-immune cells in response to tissue injury (Liew *et al*., 2016) and is also an activator of the NF-κB signaling pathway (Miller *et al*., 2011). Recently, a study showed that *Il33* mRNA was not detectable in *in vivo* testes with sc-RNA-Seq strategy but was upregulated in interstitial somatic cells of *in vitro* cultured testes (Suzuki *et al*., 2021), supporting the inflammatory response occurring in non-immune cells. In addition, they found an upregulation of *Il33* in somatic cells of *in vitro* cultured testes. IL-33 activates inflammatory responses in cells expressing ST2L receptors, such as T cells, macrophages, mast cells, and innate lymphoid cells (Lu *et al*., 2015); therefore, IL-33 may also be involved in the inflammatory response in *in vitro* cultured testes. *Ccn5* gene encodes a member of the WNT1 inducible signaling pathway (WISP) protein subfamily, which belongs to the connective tissue growth factor (CTGF) family who are characterized by four conserved domains including an insulin-like growth factor-binding domain. Interestingly, testicular cortisol and WISP2 were involved in estrogen-regulated Sertoli cell proliferation (Berger *et al*., 2019). *Cx3cr1* is a gene associated with chemokine receptor activity. The protein is involved in the migration of circulating monocytes to non-inflamed tissues where they differentiate into macrophages and dendritic cells and acts as a negative regulator of angiogenesis, probably by promoting macrophage chemotaxis (Lokka *et al*., 2020).

• ***In vitro* D16 *vs. in vitro* D4.** The role of testis-specific *1700123L14Rik* gene (*Nup50B*) in spermatogenesis is currently unknown but its presence has already been reported by several studies within the mice testicular tissue and could be related to pachytene spermatocytes I (Wang *et al*., 2016). It has been previously reported that a *Nup50* knockout mouse is not viable but that fibroblasts derived from mouse embryos can be kept in culture (Smitherman *et al*., 2000). Although referred to as a pseudogene, *1700123L14Rik* is effectively expressed as indicated in the expression atlas (Papatheodorou *et al*., 2020) and the mouse genome database (Bult *et al*., 2019) and has been previously named *Nup50rel* (Smitherman *et al*., 2000). Nup50B might substitute the canonical *Nup50* paralog at least in mouse fibroblasts. Together, these data indicate that *Nup50* has a crucial function in nuclear pore complexes assembly during mitotic exit (Holzer *et al*., 2021). In mice, *Tdrd6* is known to be gametogenesis stage- and male-specific expressed, essential for spermiogenesis, directly in interaction with the chromatoid bodies structure components Mili and Miwi (Vasileva *et al*., 2009), and mediates early steps of spliceosome maturation in primary spermatocytes, more specifically in prophase I (Akpınar *et al*., 2017). *Adam32* is a gene predominantly expressed in the testis coding to sperm surface membrane protein that is involved in sperm-egg plasma membrane adhesion and fusion during fertilization (Nishimura *et al*, 2007; Choi *et al*., 2003). *Crisp2* (*Tpx-1*) is a gene specifically expressed in male haploid germ cells and associated with the regulation of some ion channels' activity and thereby regulate calcium fluxes during sperm capacitation and the acrosome reaction (Busso *et al*., 2007). *Catspere1* encodes a subunit of the pH-dependent CatSper calcium channel, a complex involved in Sperm cell hyperactivation and sperm motility (Chung *et al*., 2017). Despite its unknown functions,

frontiers

Mroh4 shows testis-specific expression in EST Profile (UniGene) and Gene Expression Atlas (EMBLEBI). Within testicular cell types, *Mroh4* is enriched in spermatocytes and spermatids in mice (Zhou *et al*., 2017). *Gm21269* gene encodes the protein Gm21269, localized in the cytoplasm and nuclei of pachytene spermatocyte I during meiosis (Chadourne *et al*., 2021). *Abcc12* (*Mrp9*) is a member of the superfamily of ATP-binding cassette transporters. In mouse and boar sperm, MRP9 protein is clearly and exclusively localized in the sperm midpiece (Ono *et al*., 2007).

• ***In vitro* D30 *vs. in vitro* D16.** *Lcn2* encodes a protein that belongs to the lipocalin family that transport small hydrophobic molecules such as lipids, steroid hormones and retinoids (Kang *et al*., 2017; De La Chesnaye *et al*., 2020). *Tnp2* plays a key role in the replacement of histones to protamine in the elongating spermatids of mammalsin condensing spermatids, loaded onto the nucleosomes, where it promotes the recruitment and processing of protamines, which are responsible for histone eviction (Meistrich *et al*., 2003). *Oxct2b* is a haploid-specific gene regulated by a CRE-like element and bound to a testis-specific CREM isoform (Somboonthum *et al*., 2005). In mice, the transcription of *Prm1* and *Prm2* is known to initiates shortly after the completion of meiosis in round spermatids (Mali *et al*., 1989) and ceases about a week later in early elongated spermatids when all transcription stops (Kierszenbaum and Tres, 1975). Protamines substitute for histones in the chromatin of sperm during the haploid phase of spermatogenesis; they compact sperm DNA into a highly condensed, stable and inactive complex (Bao and Bedford, 2016). *Ccl8* is a chemokine gene coding to a protein involved in immunoregulatory and inflammatory processes (Van Coillie *et al*., 1999). *Tssk6* is a gene coding for a serine/threonine protein kinase that is required for postmeiotic chromatin remodeling and male fertility (Spiridonov *et al*., 2005). *Fam71a* is a testis-specific gene. As development stages progress, the number of gene transcripts increases and are at highest expression levels in adults. The action of the protein is not well known but, during, gene transcript levels of FAM71D is located in sperm flagella, increase dramatically throughout spermatogenesis and development of the testis, and is functionally involved in sperm motility (Ma *et al*., 2017). *Rps3a3* is a pseudogene coding for the ribosomal protein S3A3.

Specific References for Fig. S4

Akpınar, M., Lesche, M., Fanourgakis, G., Fu, J., Anastassiadis, K., Dahl, A. and Jessberger, R. (2017). TDRD6 mediates early steps of spliceosome maturation in primary spermatocytes. PLoS Genet. 13(3), e1006660.

Bao, J. and Bedford, M.T. (2016). Epigenetic regulation of the histone-to-protamine transition during spermiogenesis. Reproduction. 151(5), R55-70.

Berger, T., Sidhu, P., Tang, S. and Kucera, H. (2019). Are testicular cortisol and WISP2 involved in estrogen-regulated Sertoli cell proliferation? Anim Reprod Sci. 207, 44-51.

Bult, C.J., Blake, J.A., Smith, C.L., Kadin, J.A., Richardson, J.E and Mouse Genome Database Group. (2019). Mouse Genome Database (MGD) 2019. Nucleic Acids Res. 47(D1), D801-D806.

Choi, I., Woo, J.M., Hong, S., Jung, Y.K., Kim, D.H. and Cho, C. (2003). Identification and characterization of ADAM32 with testis-predominant gene expression. Gene. 304, 151-62.

Busso, D., Goldweic, N.M., Hayashi, M., Kasahara, M. and Cuasnicú, P.S. (2007). Evidence for the involvement of testicular protein CRISP2 in mouse sperm-egg fusion. Biol Reprod. 76(4), 701-708.

De La Chesnaye, E., Manuel-Apolinar, L., Damasio, L., Castrejón, E., López-Ballesteros, R., Revilla-Monsalve, M.C., Mali, P., Kaipia, A., Kangasniemi, M., Toppari, J. et al. (1989). Stage-specific expression of nucleoprotein mRNAs during rat and mouse spermiogenesis. Reprod Fertil Dev. 1(4), 369-382.

Méndez, JP. (2020). The gonadal expression pattern of lipocalin-2 and 24p3 receptor is modified in the gonads of the offspring of obese rats. Mol Med Rep. 22(2), 1409-1419.

Chung, J.J., Miki, K., Kim, D., Shim, S.H., Shi, H.F., Hwang, J.Y., Cai, X., Iseri, Y., Zhuang, X. and Clapham, D.E. (2017). CatSperζ regulates the structural continuity of sperm Ca2+ signaling domains and

frontiers

is required for normal fertility. Elife. 6, e23082.

De La Chesnaye, E., Manuel-Apolinar, L., Damasio, L., Castrejón, E., López-Ballesteros, R., Revilla-Monsalve, M.C. and Méndez, J.P. (2020). The gonadal expression pattern of lipocalin-2 and 24p3 receptor is modified in the gonads of the offspring of obese rats. Mol Med Rep. 22(2), 1409-1419.

Holzer, G., De Magistris, P., Gramminger, C., Sachdev, R., Magalska, A., Schooley, A., Scheufen, A., Lennartz, B., Tatarek-Nossol, M., Lue, H. et al. (2021). The nucleoporin Nup50 activates the Ran guanine nucleotide exchange factor RCC1 to promote NPC assembly at the end of mitosis. EMBO J. 40(23), e108788.

Janeway, C.A., Travers, P., Walport, M. and Shlomchik, M.J. (2001). The complement system and innate immunity. Immunobiology: The Immune System in Health and Disease. New York: Garland Science.

Kang, Z., Qiao, N., Tan, Z., Tang, Z. and Li, Y. (2017). Expression patterns and changes of the LCN2 gene in the testes of induced cryptorchidism and busulfan-treated mice. Syst Biol Reprod Med. 63(6), 364-369.

Liew, F.Y., Girard, J.P. and Turnquist, H.R. (2016). Interleukin-33 in health and disease. Nat Rev Immunol. 16(11), 676-689.

Lokka, E., Lintukorpi, L., Cisneros-Montalvo, S., Mäkelä, J.A., Tyystjärvi, S., Ojasalo, V., Gerke, H., Toppari, J., Rantakari, P. and Salmi, M. (2020). Generation, localization and functions of macrophages during the development of testis. Nat Commun. 11(1), 4375.

Lu, J., Kang, J., Zhang, C. and Zhang, X. (2015). The role of IL-33/ST2L signals in the immune cells. Immunol Lett. 164(1), 11-17.

Lue, Y., Wang, C., Cui, Y., Wang, X., Sha, J., Zhou, Z., Xu, J., Wang, C., Hikim, A.P. and Swerdloff, RS. (2009). Levonorgestrel enhances spermatogenesis suppression by testosterone with greater alteration in testicular gene expression in men. Biol Reprod. 80(3), 484-492.

Ma, Q., Li, Y., Luo, M., Guo, H., Lin, S., Chen, J., Du, Y., Jiang, Z. and Gui, Y. (2017). The expression characteristics of FAM71D and its association with sperm motility. Hum Reprod. 32(11), 2178-2187.

Meistrich, M.L., Mohapatra, B., Shirley, C.R. and Zhao, M. (2003). Roles of transition nuclear proteins in spermiogenesis. Chromosoma. 111(8), 483-488.

Miller A.M. (2011). Role of IL-33 in inflammation and disease. J Inflamm (Lond). 8(1), 22.

Nishimura, H., Myles, D.G. and Primakoff, P. (2007). Identification of an ADAM2-ADAM3 complex on the surface of mouse testicular germ cells and cauda epididymal sperm. J Biol Chem. 282(24), 17900-17907.

Ono, N., Van der Heijden, I., Scheffer, G.L., Van de Wetering, K., Van Deemter, E., De Haas, M., Boerke, A., Gadella, B.M., De Rooij, D.G., Neefjes, J.J. et al. (2007). Multidrug resistance-associated protein 9 (ABCC12) is present in mouse and boar sperm. Biochem J. 406(1), 31-40.

Papatheodorou, I., Moreno, P., Manning, J., Fuentes, A.M., George, N., Fexova, S., Fonseca, N.A., Füllgrabe, A., Green, M., Huang, N. et al. (2020). Expression Atlas update: from tissues to single cells. Nucleic Acids Res. 48(D1), D77-D83.

Sipione, S., Simmen, K.C., Lord, S.J., Motyka, B., Ewen, C., Shostak, I., Rayat, G.R., Dufour, J.M., Korbutt, G.S., Rajotte, R.V. et al. (2006). Identification of a novel human granzyme B inhibitor secreted by cultured sertoli cells. J Immunol. 177(8), 5051-5058.

Smitherman, M., Lee, K., Swanger, J., Kapur, R. and Clurman, B.E. (2000). Characterization and targeted disruption of murine Nup50, a p27(Kip1)-interacting component of the nuclear pore complex. Mol Cell Biol. 20(15), 5631-5642.

Somboonthum, P., Ohta, H., Yamada, S., Onishi, M., Ike, A., Nishimune, Y. and Nozaki, M. (2005). cAMP-responsive element in TATA-less core promoter is essential for haploid-specific gene expression

frontiers

in mouse testis. Nucleic Acids Res. 33(10), 3401-3411.

Spiridonov, N.A., Wong, L., Zerfas, P.M., Starost, M.F., Pack, S.D., Paweletz, C.P. and Johnson G.R. (2005). Identification and characterization of SSTK, a serine/threonine protein kinase essential for male fertility. Mol Cell Biol. 25(10), 4250-4261.

Suzuki, T., Abe, T., Ikegaya, M., Suzuki, K., Yabukami, H., Sato, T., Komeya, M. and Ogawa, T. (2021). Global inflammatory response in in vitro organ cultured testes using single-cell RNA-sequencing. [This article is a preprint and has not been certified by peer review]

Van Coillie, E., Van Damme, J. and Opdenakker, G. (1999). The MCP/eotaxin subfamily of CC chemokines. Cytokine Growth Factor Rev. 10(1), 61-86.

Vasileva, A., Tiedau, D., Firooznia, A., Müller-Reichert, T. and Jessberger, R. (2009). Tdrd6 is required for spermiogenesis, chromatoid body architecture, and regulation of miRNA expression. Curr Biol. 19(8), 630-639.

Wang, L., Guo, Y., Liu, W., Zhao, W., Song, G., Zhou, T., Huang, H., Guo, X. and Sun, F. (2016). Proteomic Analysis of Pachytene Spermatocytes of Sterile Hybrid Male Mice. Biol Reprod. 95(3), 52.

Zhou, L., Canagarajah, B., Zhao, Y., Baibakov, B., Tokuhiro, K., Maric, D. and Dean, J. (2017). BTBD18 Regulates a Subset of piRNA-Generating Loci through Transcription Elongation in Mice. Dev Cell. 40(5), 453-466.e5.

frontiers
