# Supplementary material for: Throughout in vitro first spermatogenic wave: Next-generation sequencing gene expression patterns of fresh and cryopreserved prepubertal mice testicular tissue explants
Source: Front Endocrinol (Lausanne). 2023 Mar 17;14:1112834. doi: 10.3389/fendo.2023.1112834 (PMC10063980; doi:10.3389/fendo.2023.1112834)
Supplement: Supplementary file 5 [file DataSheet_5.pdf]

Supplementary Table S1. Summary table of antibodies and fluorochromes used in the current study.

Category	Antibodies	Host	Dilution	Source	Cat# No.
	Anti-STRA8	Rabbit	1:1000	Abcam	ab49602
	Anti-rabbit (biotin-conjugated)	Goat	1:200	Abcam	ab150113
Control IgGs		Rabbit	1:200	Invitrogen™	02-6102
Alexa Fluor® 594-conjugated streptavidin		–	1:200	Invitrogen™	S32356

frontiers
